# Self-synergistic effect of Prussian blue nanoparticles for cancer therapy: driving photothermal therapy and reducing hyperthermia-induced side effects

**DOI:** 10.1186/s12951-021-00819-2

**Published:** 2021-05-04

**Authors:** Xue Xie, Wei Gao, Junnian Hao, Jianrong Wu, Xiaojun Cai, Yuanyi Zheng

**Affiliations:** 1 Shanghai Institute of Ultrasound in Medicine, Shanghai Jiao Tong University Affiliated Sixth People’s Hospital, Shanghai, 200233 People’s Republic of China; 2 Chongqing Key Laboratory of Ultrasound Molecular Imaging, Ultrasound Department of the Second Affiliated Hospital of Chongqing Medical University, Chongqing, 400010 People’s Republic of China

**Keywords:** Photothermal therapy, Inflammation, Reactive oxygen species, Prussian blue

## Abstract

**Background:**

Photothermal therapy (PTT), involving application of localized hyperthermia to kill cancer cells, has attracted wide attention in cancer therapy. The production of reactive oxygen species (ROS) during PTT may cause irreversible damage to healthy tissues around the tumor. Simultaneously, hyperthermia can stimulate inflammatory response, thus promoting tumor recurrence and metastasis. Therefore, it is of paramount importance to reduce the undesired side effects for further development of PTT.

**Results:**

Using a hydrothermal method, spherical Prussian blue nanoparticles (PBs) with uniform size were prepared. The PBs exhibited good dispersion and stability in saline with an average hydrodynamic size of 110 nm. The prepared PBs had a high photothermal conversion efficiency and photothermal stability. The PBs showed intrinsic ROS scavenging properties in vitro. Antioxidant and anti-inflammatory effects of PBs were also observed in vivo. Assessment of toxicity and endoplasmic reticulum stress-inducing ability showed that PBs did not induce an inflammatory response. Tissues of major organs of mice stained with hematoxylin–eosin showed no significant damage, indicating good biocompatibility and safety of PBs.

**Conclusion:**

The designed single-component PBs with intrinsic ROS scavenging and anti-inflammatory properties could avoid inflammatory response and heat stress-induced ROS during PTT. Thus, further research on PBs is worthwhile to achieve their clinical translation and promote the development of PTT.
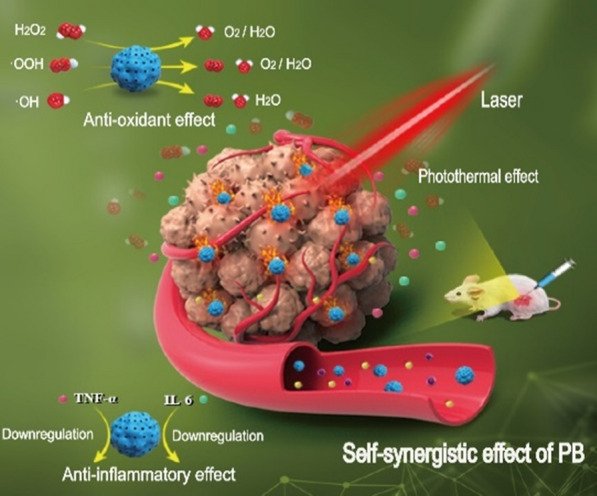

## Introduction

Photothermal therapy (PTT) is a non-invasive anticancer treatment, which has yielded good results in some clinical trials [1, 2]. PTT utilizes photothermal conversion agents to generate localized hyperthermia to cause cancer cell death mainly via apoptosis and/or necrosis in the tumor. Compared with the current clinical treatment (such as the radiotherapy, chemotherapy, surgery, etc), photothermal therapy has its unique advantages, including noninvasiveness, deep tissue penetration and spatiotemporal selectivity [3–5]. However, when PTT induces necrosis of cancer cells, there is destruction of plasma membrane integrity to cause the release of damage-associated molecular patterns and inflammatory cytokines, such as tumor necrosis factor (TNF)-α, interleukin (IL)-1β, and IL-6 [6]. The pro-survival genes in residual cancer cells can be activated by these inflammatory cytokines, inducing resistance to subsequent treatment [7, 8]. Furthermore, these inflammatory cytokines prime neutrophils to migrate to the inflamed tumor, simulating tumor regeneration and increasing resistance to the treatment [9, 10]. The overproduced ROS generated during the heat diffusion will threaten the cells and healthy tissues nearby [11–13]. Therefore, it is essential to develop efficient strategies to reduce the undesired side effects and achieve improved therapeutic efficiency of tumors.

To circumvent the above problems, photothermal conversion agents with anti-inflammatory effects may be of great significance for the management of aggressive cancers. Based on this strategy, the anti-inflammatory prodrug pyrene-aspirin was loaded on gold nanorod-encapsulated graphitic nanocapsules to achieve photothermal ablation of the tumor and simultaneously alleviate PTT-triggered inflammation [14]. This photothermal-anti-inflammatory strategy overcomes the undesired induction of proinflammatory cytokines during the process of PTT. Similarly, with the aim of attenuating the inflammatory response during PTT, a CO nanogenerator was constructed with partially oxidized tin disulfide nanosheets, a tumor-targeting polymer (PEG-cRGD), and the chemotherapeutic drug doxorubicin. In this system, CO was generated from CO_2_ via the photocatalytic reduction in the presence of partially oxidized tin disulfide nanosheets. This process not only increased the chemotherapeutic effect of doxorubicin but also reduced the PTT-induced inflammation [15]. Such a dual-functional strategy may scavenge heat stress-induced ROS or their precursors, which protects normal or untreated cells from oxidative damage during PTT. Based on this concept, gold nanorods coated with a platinum shell (PtAuNRs) were designed as an efficient ROS scavenger and photothermal conversion agent. This PtAuNRs system showed high photothermal efficiency and ROS-scavenging property, which could be employed to effectively treat cancer cells as they induced hyperthermia but simultaneously reduced PTT-induced ROS generation [16]. These reported multi-component systems involved a complicated synthesis process and high cost [17]. More importantly, only few studies have focused on both inflammation and ROS during PTT.

**Fig. 1:**
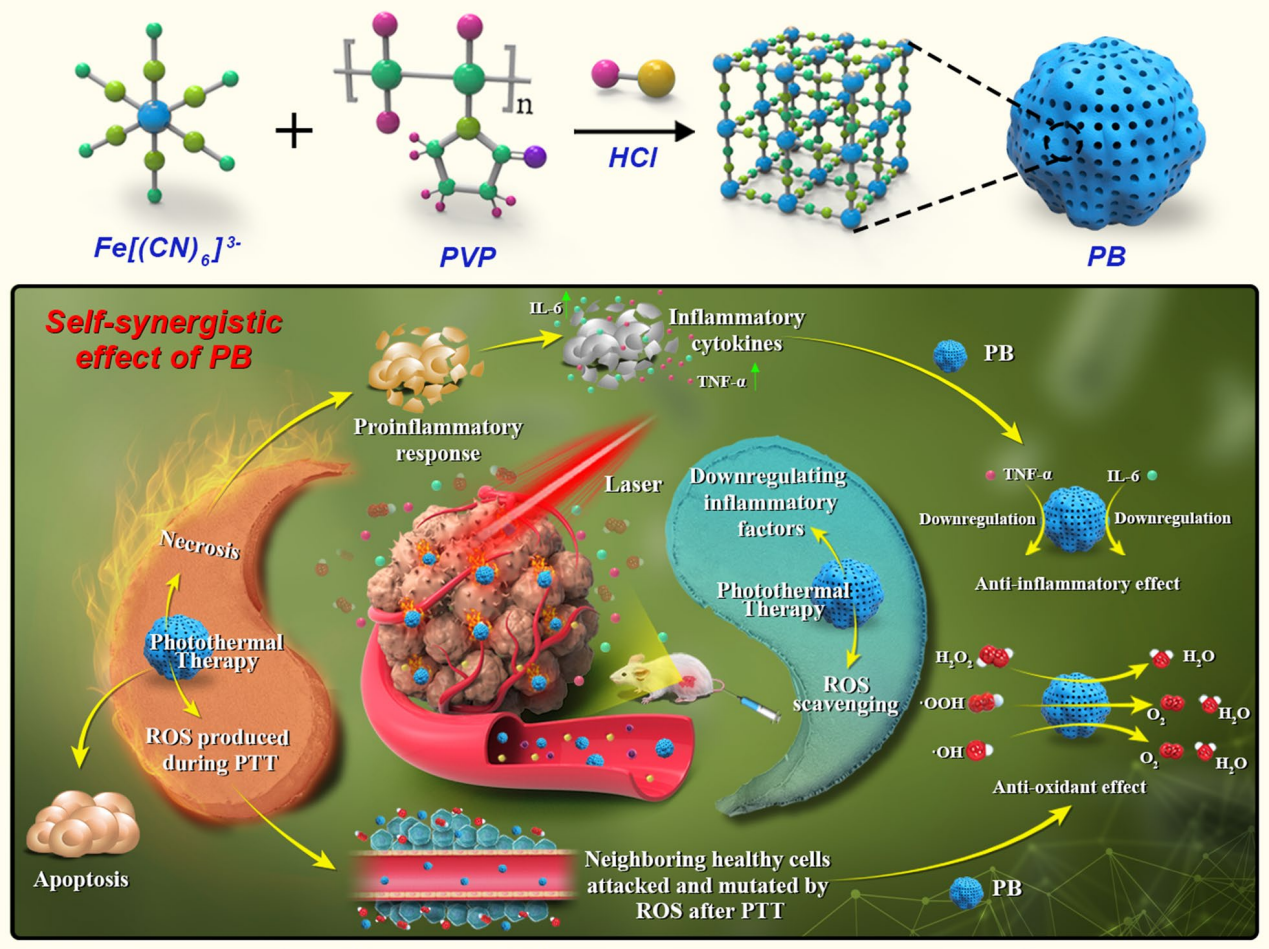
Schematic illustration of the self-synergistic effect of PBs. Single-component nanomaterials utilize their intrinsic properties to enhance the overall treatment effect while reducing the side effects. Owing to their anti-inflammatory effect, PBs downregulate the levels of inflammatory cytokines including IL-6 and TNF-α. PBs also have an antioxidant property, which converts harmful ROS (such as ·OH, ·OOH, and H_2_O_2_) into harmless H_2_O and O_2_. Furthermore, when activated by laser irradiation, PBs exert a photothermal effect against tumors. The designed PBs with intrinsic properties overcome the problem of inflammatory response and heat stress-induced ROS during PTT

Herein, based on the principle of "simpler is better", the concept of "self-synergistic effect of nanomaterials" is proposed, that is, single component nanodrugs take full use of their inherent characteristics to enhance each other's positive effect and/or reduce side effect. In this study, the concept of "self-synergistic effect of nanomaterials" is discussed with Prussian blue nanoparticles (PBs) as an example (Fig. 1). PBs showed good photothermal conversion, ROS scavenging, and anti-inflammatory properties. Their intrinsic ROS scavenging property prevents ROS generation due to heat stress associated with PTT. Simultaneously, the injected PBs in blood circulation downregulated the inflammatory cytokines including IL-6 and TNF-α via their intrinsic anti-inflammatory properties (Fig. 1). This efficient strategy integrates the photothermal-antioxidant and the photothermal-anti-inflammatory properties. The discovery of self-synergistic effect of PBs promotes their further clinical translation. More importantly, the concept of self-synergistic effect encourages scientist to further explore the intrinsic properties of nanomaterials for enhancing their positive effects and/or reducing side effects.

## Materials and methods

### Preparation of PBs

K_3_[Fe(CN)_6_] (495 mg), PVP (5 g), and HCl solution (1 M, 40 mL) were mixed in a glass bottle or reaction vessel under magnetic stirring for about 30 min until clear. Then the vial was placed in an electric oven at 80 °C for 24 h. PBs were obtained after centrifugation (20,000 r/min, 60 min, 4 ℃) and washing the residue with deionized water several times. They were then re-dispersed in 0.9% saline and stored at room temperature until subsequent use.

### Characterization of PBs

The microstructure of the nanoparticles was observed by using JEM-2100F transmission electron microscope (TEM) and scanning electron microscope (SEM). The concentration of PBs (Fe concentration) was determined by inductively coupled plasma atomic emission spectroscopy (Agilent Technologies, USA). The hydrodynamic particle size of PBs was determined by dynamic laser scattering (DLS) on a ZetaSizer system (Nano ZS90, Malvern Instruments Ltd). The composition and crystal properties of the samples were tested by using X-ray diffraction (XRD) patterns on a Rigaku D/MAX-2200PX X-ray diffraction system. The chemical status of the samples was characterized by X-ray photoelectron spectroscopy (XPS) on ESCAlab250 (Thermal Science Company). Fourier transform infrared spectroscopy (FT-IR) was used for the analysis of chemical bonds. The ultraviolet-visible-near-infrared (UV–vis-NIR) absorption spectrum was recorded by using a Shimadzu UV-3600 spectrophotometer.

### Photothermal performance of PBs

The photothermal performance of the aqueous solution of PBs with different Fe concentrations was studied by monitoring the temperature changes under an 808 nm high-power multimode pump laser (Shanghai Connect Fiber Optics Company). Pure water was chosen as the control. The real-time temperature changes and thermal images of the irradiated aqueous dispersion were recorded by an FLIR thermal camera (FLIR Thermal CAM E40) and FLIR Examiner software. In addition, the temperature increases of the PBs aqueous solution (100 μg mL^−1^ and 200 μg mL^−1^) irradiated by the 808 nm laser at different power intensities (0.2, 0.4, 0.8, and 1.0 W cm^−2^) was tested. To evaluate the stability of PBs after irradiation, the aqueous solutions (100 μg mL^−1^) were irradiated by the 808 nm laser for five times and for 10 min each and then subjected to DLS and UV–vis-NIR absorption spectrum analysis.

The photothermal conversion efficiency of PBs was ascertained using the literature procedure and calculated using the following equation [20].

$$\eta =\frac{hS({T}_{max}-{T}_{amb})-{Q}_{0}}{I(1-{10}^{-A})}$$

H and S represent the heat transfer coefficient and surface area. T_max_ and T_amb_ is the equilibrium temperature and ambient temperature, respectively. Q_0_ is the heat absorption of the quartz container, I is the laser power density, and A is the absorbency of the PBs at 808 nm.

### In vitro ROS scavenging effects of PBs

The TiO_2_/UV system was used to test the scavenging effect of PBs on ·OH. Electron spin resonance (ESR, Bruker EMX spectrometer) was used to detect ·OH generation in the form of 5,5-Dimethyl-1-Pyrrolidine-N-oxide (DMPO)/·OH spin adduct. TiO_2_ suspensions were exposed to UV light (340 nm) to produce ·OH radicals, and different concentrations of PBs solution (12.5, 25, 50, and 100 μg mL^−1^) were added into 0.1 mg mL^−1^ TiO_2_ (P25, Degussa Huls Corporation, Germany) and 50 mM DMPO. ESR spectra were recorded after 5 min of exposure to UV light. To verify the superoxide anion (·OOH) scavenging ability of PBs, xanthine and xanthine oxidase were mixed in a buffer solution (pH 5.0) to generate ·OOH, then DMPO was used to trap the ·OOH in the form of DMPO/·OOH spin adduct. The catalase-like activity of PBs was determined by detecting the oxygen production with an oxygen electrode on Multi-Parameter Analyzer (DZS-708, Cany, China). For this, 1.5 mL H_2_O_2_ solution (10 mM) was added to 13.5 mL PBs solution (100 μg mL^−1^) under neutral pH conditions. The generated soluble O_2_ (unit: mg L^−1^) was measured at different reaction times.

### Cell culture and in vitro cytotoxicity assay

4T1 murine breast cancer cells (Shanghai institute of Cells, Chinese Academy of Sciences) were cultured in Dulbecco’s Modified Eagle’s Medium (DMEM, high glucose, GIBCO, Invitrogen), which were supplemented with 10% fetal bovine serum (FBS) and 1% penicillin/streptomycin at 37 °C under 5% CO_2_ atmosphere. For in vitro cytotoxicity assays, a standard CCK-8 viability assay (Gibco, Shanghai) was conducted. 4T1 cells were pre-cultured into a 96-well plate at a density of 1 × 10^4^ cells/well for 24 h. Then, the 96-well plate culture medium was removed and rinse it twice with phosphate buffer (PBS). Subsequently, PBs dispersion with different concentrations (0–400 μg mL^−1^) was dispersed into the 10% FBS containing DMEM high-glucose medium. Then, the fresh medium containing PBs was added into each well and co-incubated for another 12, 24 and 48 h, respectively. Then, the cells were washed with PBS and the standard CCK-8 assay (100 μL, V_CCK8_: V_DMEM_ = 1:9) was performed to test the cell viabilities, which were measured on a microplate reader at a wavelength of 450 nm after about 30–60 min.

### In vitro photothermal effect of PBs against cancer cells

4T1 cells were pre-seeded in 96-well plates (1 × 10^4^ cells/well) in DMEM containing 10% FBS overnight to allow the attachment of cells. PBs was dispersed with DMEM to form different concentrations of dispersion (0, 12.5, 25, 50, 75, 100, 200 μg mL^−1^) and then inoculated into the 96-well plate instead of the old medium. After being cultured for 4 h, part of cells was irradiated by an 808 nm laser at 1 W cm^−2^ for 10 min. Meanwhile, PBs was incubated with 4T1 cells at a final concentration of 100 μg mL^−1^ for 4 h and then subjected to 10 min laser irradiation at different power densities before incubation was continued for a total time of 24 h. After that, the culture medium was removed in all wells and cells were rinsed with PBS for three times. A standard CCK-8 assay was used to measure cell viabilities. Cell viabilities were additionally evaluated with a calcein-AM/PI cell staining assay. After subjecting 4T1 cells to the various treatments detailed above, the cells were stained with calcein-AM (10 μL) and PI solutions (15 μL, Dojindo Molecular Technologies, Inc.) in PBS for 15 min in the dark. The cells were washed with PBS three times, and subsequently visualized using confocal laser scanning microscopy (CLSM, FV1000, Olympus Company, Japan).

### Intracellular endocytosis of PBs by CLSM observation

CLSM was used to evaluate the uptake of PBs by cancer cells. To obtain PBs-FITC, PBs was stirred with fluorescein isothiocyanate (FITC, 5 mg, Sigma-Aldrich, Shanghai, China) at room temperature overnight in dark. The final product was washed with ethanol for several times to acquire PBs-FITC. 4T1 cells were seeded into the CLSM-specific dishes (35 mm × 10 mm, Corning Inc, New York, USA) at a density of 1 × 10^5^, and incubated for 24 h. The culture media was replaced by PBs-FITC (1 mL, 100 μg mL^−1^, dispersed into DMEM containing 10% FBS), which were then cultured for 0, 1, 2, and 4 h, respectively. The cells were washed with PBS and DAPI (100 μL, Beyotime Biotechnology) diluted with methanol (DAPI: methanol = 1:10) was added to stain cell nuclei for 15 min. The cells were washed and imaged by CLSM.

### Intracellular antioxidant stress mediated by PBs

RAW 264.7 macrophages were selected to evaluate the antioxidant and anti-inflammatory effects of PBs. RAW 264.7 macrophages were pre-cultured into a 96-well plate (1 × 10^4^ cells/well) for 24 h. Then, the culture medium was removed and rinse it twice with PBS. Subsequently, cells were treated with different formulations, including control, H_2_O_2_ (300 μM), PBs (100 μg mL^−1^), H_2_O_2_ (300 μM) + PBs (25 μg mL^−1^), H_2_O_2_ (300 μM) + PBs (50 μg mL^−1^) and H_2_O_2_ (300 μM) + PBs (100 μg mL^−1^). PBs was pretreated for 4 h and then incubated with H_2_O_2_ for another 4 h. After washed with PBS for three times, the cell cytotoxicity was determined with standard CCK-8 assay.

### Intracellular anti-inflammation effect mediated by PBs

RAW 264.7 macrophages were precultured on a 6-well plate for 24 h. After incubated with PBs for 4 h, RAW 264.7 macrophages were stimulated by lipopolysaccharide (LPS) for another 12 h. The supernatant was collected to measure proinflammatory cytokines (IL-6, TNF-α, IL-1β) by enzyme-linked immunosorbent assay (ELISA) kit (Anogen-Yesbiotech, Canada).

### In vivo serum biochemistry and routine blood test

BALB/c mice (n = 5) were intravenously administered 200 μL different concentration of PBs (0, 2 and 4 mg mL^−1^). After 24 h, the mice were anesthetized, and the spleen, heart, lung, liver, kidney, and blood were obtained. The spleen, heart, lung, liver, and kidney tissue sections were stained with hematoxylin–eosin (H&E). Serum was separated from the collected blood and assayed for alanine transaminase (ALT) and aspartate transaminase (AST) using a Sysmex XS-800i automated hematology analyzer. The levels of IL-6 and TNF-α were measured by using ELISA kit. Each experiment was carried out in triplicate.

### Quantification of immune cells in the liver and spleen

Fresh liver and spleen tissues were isolated aseptically and then placed in pre-cooled PBs and passed through a 70-μm-mesh nylon mesh placed in 2 mL precooled RPMI 1640 medium. We obtained the lymphocyte suspension by centrifugation and suspension. Then the lymphocyte suspension was transferred to a centrifuge tube. After adding 10 volumes of RPMI 1640 medium and washed once, the supernatant was discarded. PE-CD3, FITC-CD4, and Percp/cy5.5-CD8 were used to stain the CD3^+^T, CD4^+^T, and CD8^+^T cells, respectively (eBioscience Inc, San Diego, CA, USA). Flow cytometry was used to analyze the percentage of CD3^+^T, CD4^+^T, and CD8^+^T cells.

### TUNEL assay

The TUNEL assay was used to specifically detect the fragmented genomic DNA usually produced by sequential activation of caspases and endonucleases during apoptosis. Dewaxing, tissue rehydration, and staining were carried out according to the manufacturer’s instructions for the fluorescence-conjugated TUNEL assay kit (Roche, Mannheim, Germany). For counting the total number of cells in tissue samples, DAPI was added before mounting the coverslips to stain the nuclei. Images were captured with a fluorescence microscope (Olympus BX61W1with Fluoview FV1000 software, Japan) and then analyzed using the ImagePro software. Three different image areas of at least 500 cells were counted to determine the apoptosis rate.

### Histopathological examination

The tissues were fixed overnight in 10% neutral-buffered formalin, embedded in paraffin blocks, cut into 4-μm sections, and mounted onto glass slides. After H&E staining, the pathological changes in the tissues were observed under an optical microscope (Leica DM4000M, Germany) by a well-trained pathologist.

### In vivo ROS-scavenging and anti-inflammatory effects of PBs

BALB/c mice were purchased from Animal experiment Center of Shanghai Sixth People's Hospital (Animal Welfare Ethics acceptance number: DWLL2019-0309 Animal Experiment Registration number No: DWSY2018-035). All the animal procedures were performed under the protocol approved by the Institutional Animal Care and Use Committee of Shanghai Sixth People’s Hospital. All the animal experimental operations were in compliance with the National Guidelines for Animal Protection. The mice were divided into three groups (n = 5): (1) control group (saline), (2) LPS group, and (3) PBs + LPS group. PBs were intravenously injected into the mice via the tail vein. The inflammatory model was established by injection of LPS on day 7. After 24 h, the serum samples and liver tissues were taken. Hematological changes and inflammatory cytokines were detected. Then, ROS in the liver was detected by flow cytometry. The liver tissues were stained with H&E for observing the structure and morphology and subjected to the TUNEL assay for observing apoptosis and necrosis.

### In vivo PTT efficacy of PBs

To establish the 4T1 tumor xenograft, female BALB/c nude mice (4–6 weeks) were used, 4T1 cells (1 × 10^6^ cells per mouse) were suspended in 100 μL of PBS and injected into the right hip of mice. When the tumor volume reached 100 mm^3^, the mice were divided into four groups (n = 5 in each group), as follows: (1) control group (Saline), (2) laser group, (3) PBs + laser group (intravenous injection, dose of 5 mg kg ^−1^) and (4) PBs + laser group (intratumoral injection, dose of 1.25 mg kg ^−1^). After 24 h of intravenous or intratumoral injection, PTT was performed with 808 nm laser irradiation (1.0 W cm^−2^, 10 min). A thermal infrared camera was used to monitor the temperature rise in real time. The tumor size and body weight of mice were measured every two days during the observation period after treatment. Tumor volume was calculated by the following equation: width^2^ × length × 0.5. After 24 h of PTT, blood samples were taken from the mice to measure inflammatory cytokines IL-6 and TNF-α in the serum. After that, the tumors were dissected and stained with H&E for observing the structure and morphology of the tumor and with Ki-67 antibody for determining cancer cell growth. According to the standard animal protocol, euthanasia was carried out once the tumor volume reached 1000 mm^3^.

### Statistical analysis

All data are presented as mean ± standard deviation (SD), and the significance analysis between groups was conducted using the Student’s two-tailed t test (*, p < 0.05; **, p < 0.01; ***, p < 0.001; ****, p < 0.0001).

## Results and discussion

### Characterization and intrinsic properties of PBs

**Fig. 2:**
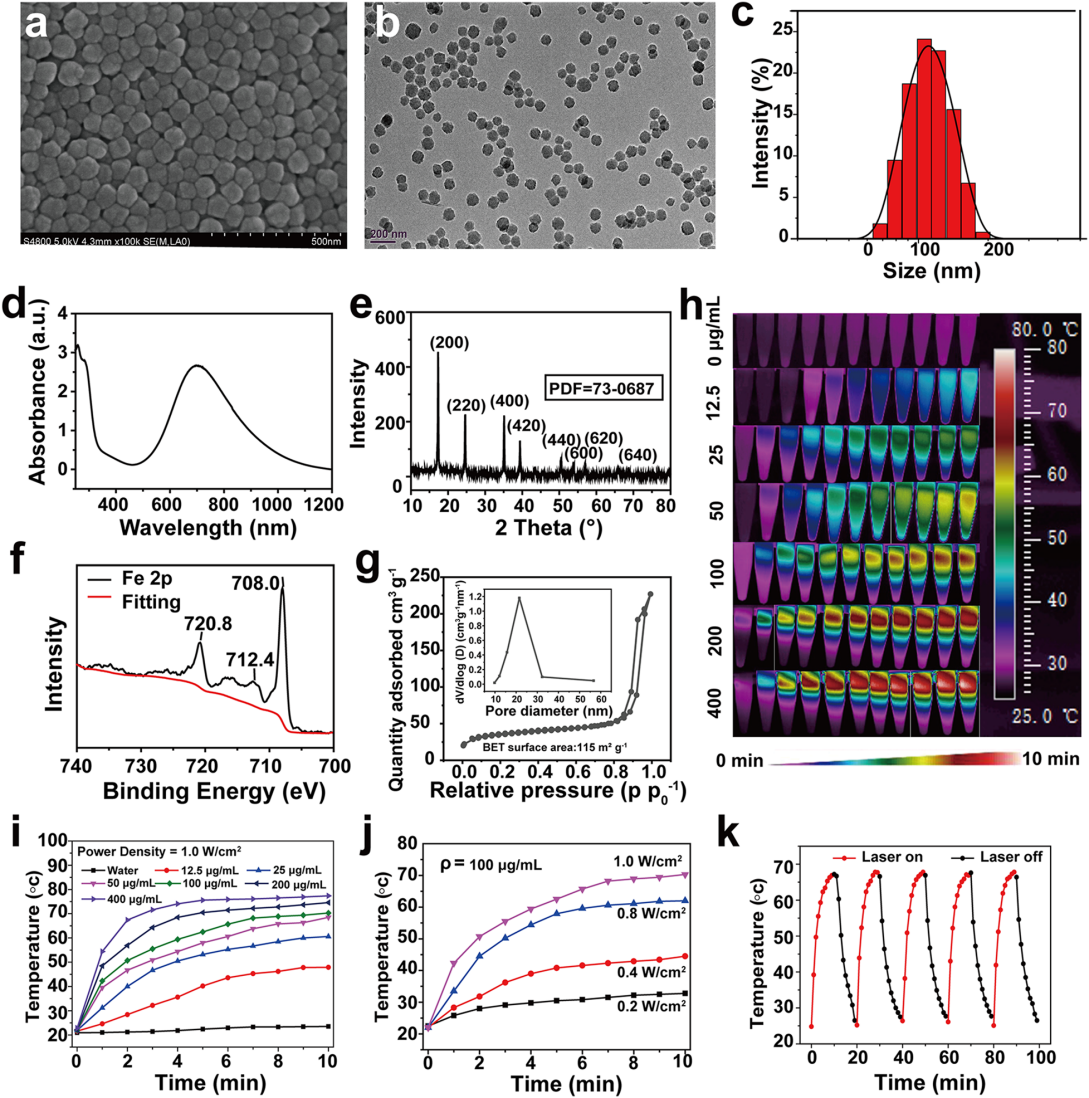
Structural and morphological characteristics and in vitro photothermal performance of PBs. **a** SEM image, **b** TEM image of highly dispersed PBs. **c** DLS size distribution analysis of PBs dispersed in aqueous solution. **d** UV–vis-NIR spectra of PBs dispersed in aqueous solution. **e** XRD patterns of PBs. **f** XPS analysis of PBs. **g** N_2_ adsorption–desorption isotherms of PBs. (Illustration: pore size distribution). **h** Infrared thermal images at elevated concentrations under 808 nm laser irradiation (1.0 W cm^−2^). **i** Photothermal heating curves of PBs at different concentrations under 808 nm laser irradiation (1.0 W cm^−2^). **j** Photothermal heating curves of PBs dispersed in aqueous solution irradiated at different power intensities (0.2, 0.4, 0.8, and 1.0 W cm^−2^). **k** Recycling heating curves of PBs dispersed in aqueous solution (100 μg mL^−1^) irradiated with five laser on/off cycles (1.0 W cm^−2^)

PBs were synthesized from PVP, K_3_[Fe (CN)_6_], and hydrochloric acid via an efficient hydrothermal synthesis strategy. SEM (Fig. 2a) and TEM images (Fig. 2b and Additional file 1: Fig. S1) display the robust spherical morphology, uniform size, and good dispersibility of PBs. The average hydrodynamic size of PBs was 110 nm (Fig. 2c). The UV–vis-NIR absorbance spectrum of PBs revealed a wide absorption in the NIR region (Fig. 2d), and the absorption peak at 700 nm could be attributed to the intermetallic charge-transfer band from Fe^II^ to Fe^III^ in the structure of PBs. XRD data (Fig. 2e) showed that the characteristic peaks of the prepared PBs matched well with those of Fe_4_[Fe(CN)_6_]_3_ (JCPDS# 73–0687). The peaks of Fe2p^3/2^ (712.4 eV) and Fe2p^1/2^ (720.8 eV) matched with those of Fe^III^ in Fe_4_[Fe(CN)_6_]_3_, and the peak at 708.0 eV represents the existence of Fe2p^3/2^ in [Fe(CN)_6_]^4−^ (Fig. 2f). FT-IR spectroscopy (Additional file 1: Fig. S2) displayed a characteristic peak around 2085 cm^−1^ of Fe^II^-CN-Fe^III^. N_2_ absorption–desorption results showed that the specific surface area of PBs was 115 m^2^ g^−1^ and the average piled pore diameter was 12 nm (Fig. 2g). We then evaluated the photothermal conversion properties of PBs at the wavelength of 808 nm. The increasing temperature profile of PBs was dependent on the concentration of PBs (Fig. 2h, Fig. 2i, and Additional file 1: Fig. S3), laser power intensity (Fig. 2j and Additional file 1: Fig. S4), and irradiation time (Fig. 2h–j and Additional file 1: Figs. S3, S4). The prepared PBs showed good photothermal stability (Fig. 2k) and high photothermal conversion efficiency (Additional file 1: Fig. S5), the photothermal conversion efficiency of PBs was calculated as 31.8%, demonstrating good photothermal conversion properties of the prepared PBs. These results are also consistent with the reported research of PBs as a photothermal conversion agent [18–20].

**Fig. 3:**
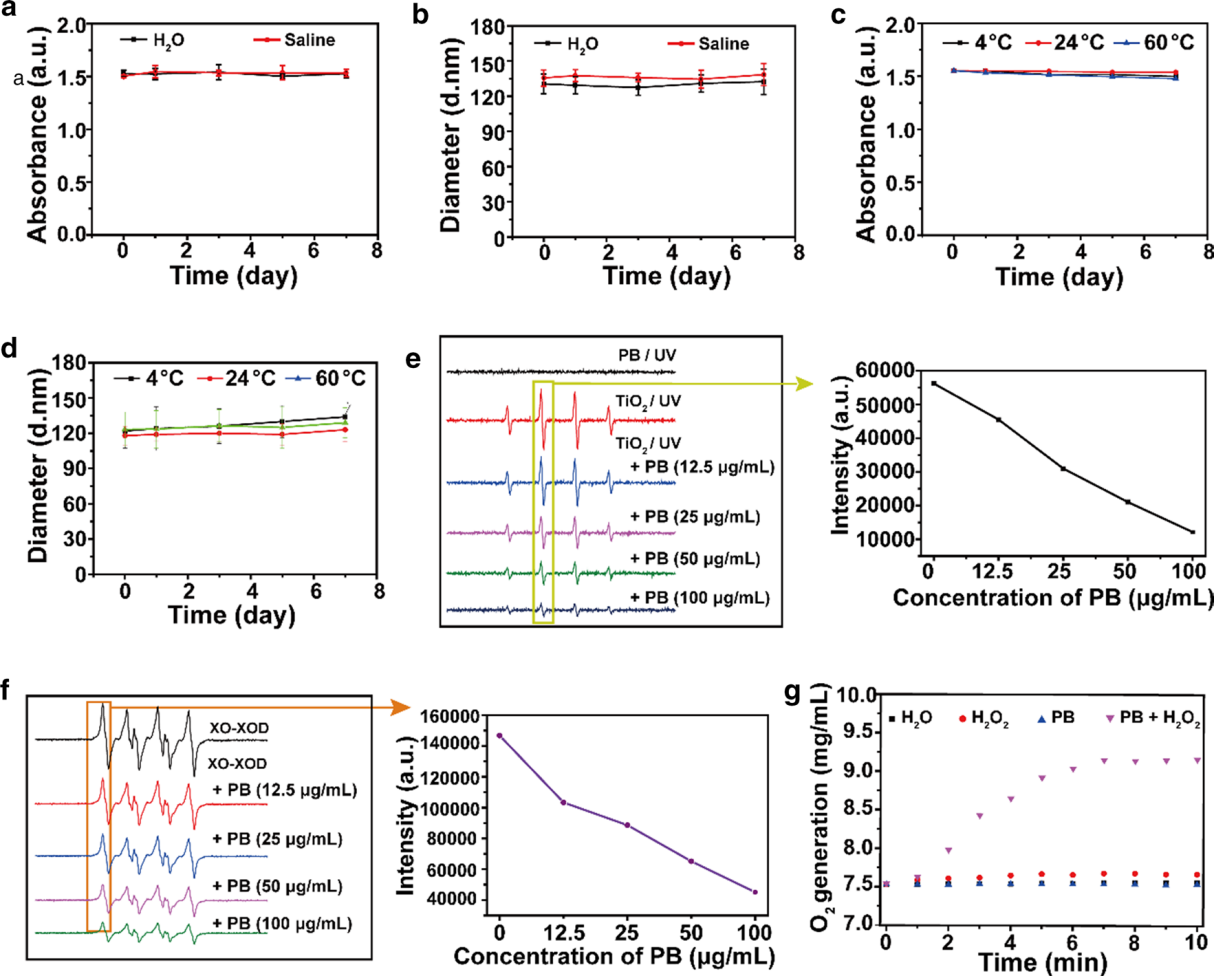
**a** Time-dependent UV–vis-NIR absorbance peak at 700 nm of PBs in water and saline. **b** Time-dependent hydrodynamic diameters of PBs in water and saline. **c** Temperature-dependent UV–vis-NIR absorbance peak at 700 nm of PBs. **d** Temperature-dependent hydrodynamic diameters of PBs. **e** Scavenging effect of PBs on ·OH generated by a TiO_2_/UV system. **f** Scavenging effect of PBs on ·OOH. **g** Catalase-like activity of PBs. PBs can be used to catalyze the decomposition of H_2_O_2_ to produce oxygen and water.

The prepared PBs showed good dispersibility and stability in water and saline (Additional file 1: Fig. S6), and the UV–vis-NIR absorbance spectra of PBs dispersions remained stable before and after 808 nm laser irradiation for 10 min and 30 min (Additional file 1: Fig. S7). There were no remarkable changes in the absorption peak and hydrodynamic diameter of PBs in water and saline at different temperatures for at least seven days (Fig. 3a–d), indicating good dispersed in water and saline and good stability in vitro. Then, we selected ·OH, ·OOH, and H_2_O_2_ as representative ROS species to investigate the ROS-scavenging properties of PBs. The prepared PBs efficiently scavenged ·OH generated from the TiO_2_/UV system (Fig. 3e), ·OOH generated from the xanthine/xanthine oxidase system (Fig. 3f), and H_2_O_2_ (Fig. 3 g). PBs showed a concentration-dependent profile of scavenging ·OH, ·OOH, and H_2_O_2_.The scavenging capability of PBs may be ascribed to the abundant redox potentials, showing peroxidase, catalase, and superoxide dismutase activity [21–23].

### In vitro cell experiments

In view of the ideal photothermal characteristics of PBs in vitro, its photothermal killing effect on cancer cells was further explored. First, the intrinsic toxicity of PBs was investigated by standard CCK-8 assay. 4T1 cells were incubated with PBs of different concentrations (0–400 μg mL^−1^) for 12, 24 and 48 h, respectively. No obvious cytotoxicity can be observed after incubation with PBs for 48 h, even at the co-incubation concentration of as high as 400 μg mL^−1^ (Additional file 1: Fig. S8), indicating the high cytocompatibility. The photothermal effect of PBs on killing cancer cells with different PBs concentrations and laser power densities were also evaluated (Additional file 1: Fig. S9). It was worth noting that the PBs + laser group showed a significant advantage in photothermal killing effect on 4T1 cancer cells, which could be more intuitively displayed from the images under CLSM (Additional file 1: Fig. S10). After continuous 808 nm laser irradiation of 10 min, a large number of dead cells (red fluorescence) were appeared in PBs + laser group. We also found that the photothermal effect was dependent on the concentration of PBs and the intensity of laser power. We further tested the intracellular uptake of FITC-labeled PBs (Additional file 1: Fig. S11). It can be found that the endocytosis process was time-dependent as proved by increased intracellular FITC-fluorescence signals at extended incubation durations (1, 2, and 4 h) (Additional file 1: Fig. S12).

We further evaluated the antioxidant and anti-inflammatory effects of PBs on RAW 264.7 macrophages. H_2_O_2_ (300 uM) was used to stimulate RAW 264.7 macrophages to induce oxidative stress. The results showed that the survival rate after treated with PBs alone was 96.7%, while reduced to 19.6% in H_2_O_2_ group. It could be found that the survival rate could be increased in the presence of different concentrations of PBs. The results showed that PBs may be able to reduce the oxidative damage of macrophages induced by H_2_O_2_ (Additional file 1: Fig. S13). We further studied the anti-inflammatory effect of PBs on RAW 264.7 macrophages stimulated by LPS. Compared with the control group, the incubation of PBs with macrophages did not cause the increase of IL-1β, IL-6 and TNF-α. The levels of these factors in the cells incubated with PBs were lower than those in the LPS group. These results confirmed that PBs could reduce the inflammation caused by LPS in vitro (Additional file 1: Fig. S14).

### Toxicity and endoplasmic reticulum (ER) stress-inducing ability of the prepared PBs

**Fig. 4:**
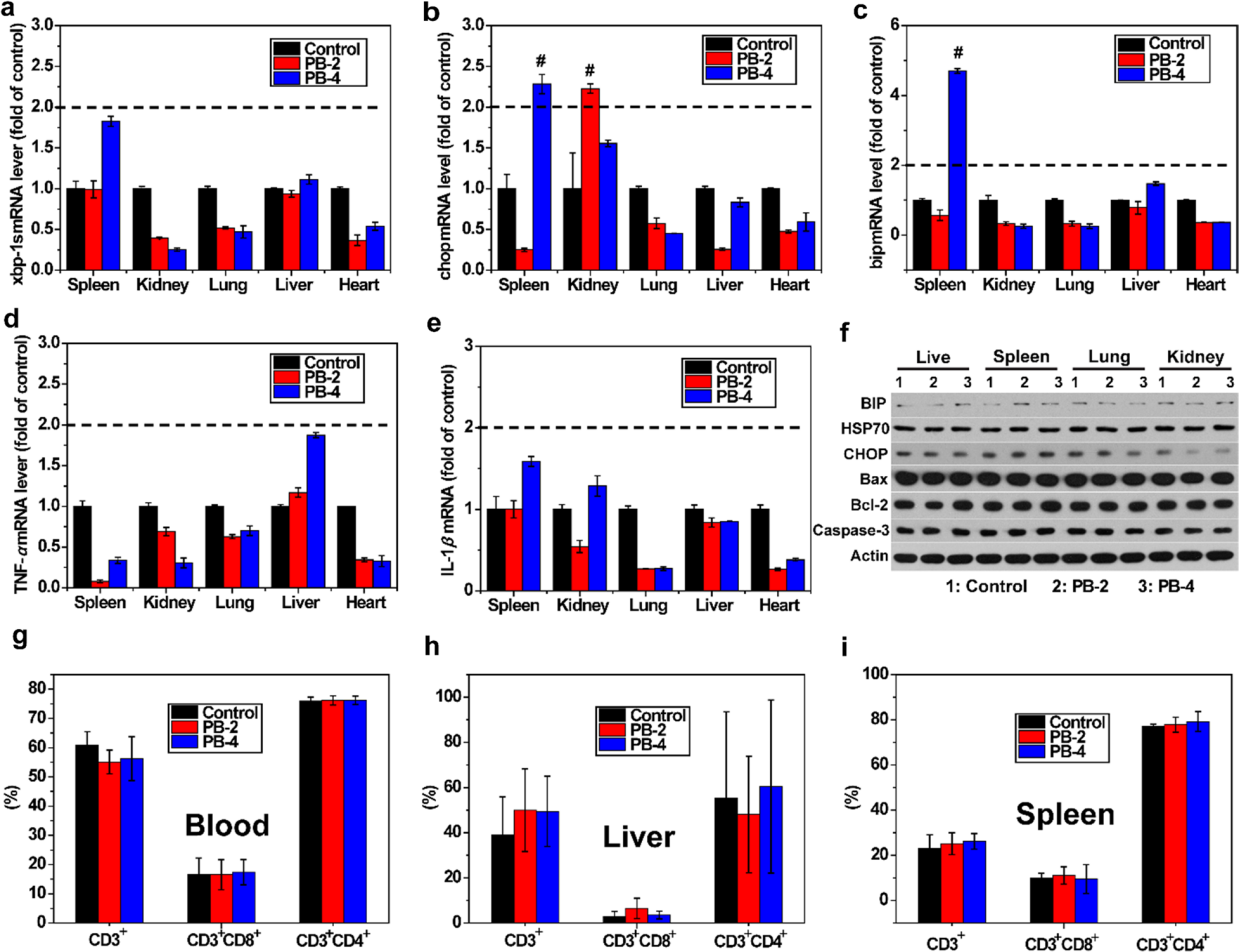
Toxicity and ER stress-inducing ability of PBs. Representative RT-PCR results from five independent replicates.** a** xbp-1 s. **b** chop. **c** bip. **d** TNF-α. **e** IL-1β. Dotted lines indicate a > twofold change in expression compared to the vehicle control group, showing significant change. #, p < 0.001 compared to the vehicle control group.** f** Expression of AOP marker proteins after 24 h treatment with PBs at doses of 2 mg mL^−1^ (PB-2) and 4 mg mL^−1^ (PB-4). Flow cytometry analysis of CD3^+^, CD3^+^CD8^+^, and CD3^+^CD4^+^ T cells in the **g** blood, **h** liver, and **i** spleen. xbp-1 s: X-box binding protein 1; chop: CCAAT-enhancer-binding protein homologous protein gene; bip: binding immunoglobulin protein gene; TNF-α: tumor necrosis factor-α; IL-1β: interleukin-1β gene; AOP: adverse outcome pathway

According to the US Environmental Protection Agency and National Research Council, toxicity testing for cellular responses or adverse outcome pathways (AOP) to evaluate adverse health effects are the preferred toxicity testing strategies [24]. We used a BALB/c mouse model to investigate the toxicity and ER stress-inducing ability of the prepared PBs [22]. BALB/c mice were administered a single intravenous injection of PBs, and the body weight, marker gene expression, hematology, inflammation, and histopathology were also evaluated. There were no significant changes in the body weight of the mice after intravenous injection of PBs (data not shown). Most of the PBs were captured by the reticuloendothelial system-related organs such as the spleen and the liver, consistent with previous reports [19, 22]. The effects of PBs on the expression of AOP markers were evaluated using reverse transcription-polymerase chain reaction (RT-PCR). Figure 4a-f represents the effects of PBs-induced ER stress responses and inflammation on different tissues. No significant upregulation of the spliced form of X-box binding protein 1 (xbp-1 s) was observed (Fig. 4a). The expression of CCAAT-enhancer-binding protein homologous protein (chop) and binding immunoglobulin protein (bip) genes was similar in various tissues, and they were overexpressed in the spleen after exposure to a high concentration of PBs (Fig. 4b, c). No obvious changes in the levels of TNF-α and IL-1β were observed in any of the tested tissues (Fig. 4d, e), indicating that the PBs could not induce an inflammatory response. In addition, there were no obvious changes in the expression levels of AOP marker proteins after 24 h of treatment with PBs (Fig. 4f). To study the immune response in the blood, liver, and spleen after injection of PBs, important effector cell types (CD3^+^, CD3^+^CD8^+^, and CD3^+^CD4^+^ T cells) were enumerated. The frequencies of CD3^+^, CD3^+^CD8^+^, and CD3^+^CD4^+^ T cells did not significantly change in the blood, liver, and spleen after injection of PBs (Fig. 4g–i, Additional file 1: Figs. S15-S17). The above results indicated that the prepared PBs are nontoxic to mice.

### ROS-scavenging effect and anti-inflammatory effect of PBs in vivo

**Fig. 5:**
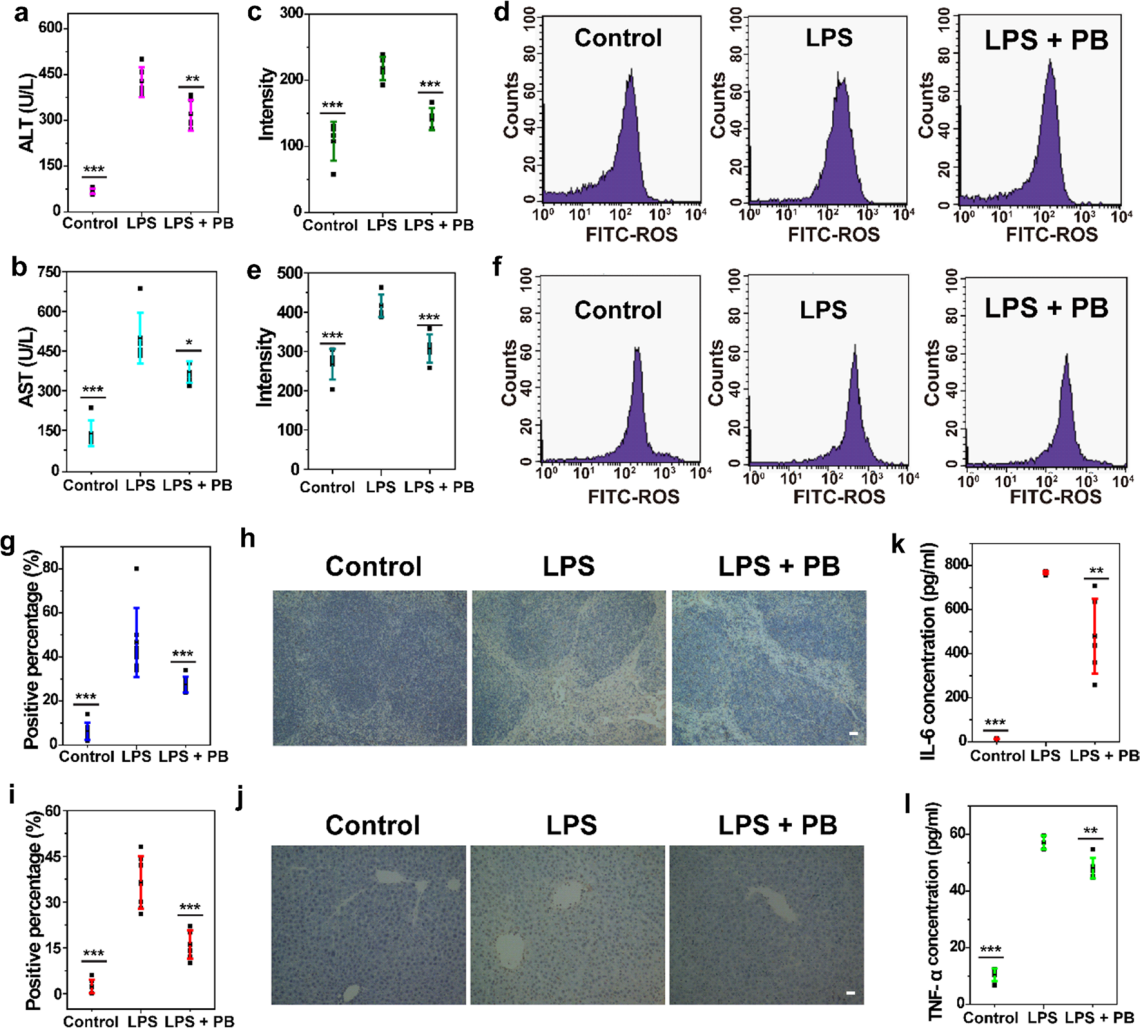
Scavenging effects of PBs on the level of ROS and inflammatory cytokines. **a** ALT. **b** AST. ROS levels in the **c**, **d** liver and **e**, **f** spleen of control, LPS-treated, and LPS + PBs-treated mice. Quantification and images of TUNEL-positive cells in the **g**, **h** liver and **i**, **j** spleen tissues from control, LPS-treated, and LPS + PBs-treated mice. Scale bar: 50 μm. **k** IL-6 and** l** TNF-α levels in the serum of control, LPS-treated, and LPS + PBs-treated mice. (*p < 0.05; **p < 0.01; ***p < 0.001)

We used a LPS-induced mouse model of inflammation to investigate the role of PBs in modulating inflammation and oxidative stress. Compared to the control group, LPS increased ROS and inflammatory cytokine expression in the serum and apoptosis in the liver and spleen, indicating successful establishment of the inflammation mouse model (Fig. 5). ALT and AST levels significantly decreased after treatment with PBs, demonstrating that PBs possessed the potential to protect hepatocytes from damage (Fig. 5a, b). ROS overproduction during inflammation can damage proteins, lipids, and DNA. PBs showed strong scavenging capability against LPS-induced increased ROS in the liver and spleen, and the ROS level in the treated group was similar with that in the control group (Fig. 5c-f). From TUNEL-staining (Fig. 5h, j), almost no apoptotic cells were observed in the control group, while it significantly increased after LPS treatment in both liver and spleen. Specifically, the apoptotic scale in group treated by LPS after PBs pretreatment was obviously smaller than those in the LPS group alone (Fig. 5h, j). The quantitative analysis of apoptotic cells in liver and spleen after different treatments further proved that PBs can protect liver and spleen by reducing LPS-induced inflammation (Fig. 5g, i). H&E staining, focal nuclear pyknosis, inflammatory cell infiltration, and even bile stasis could be observed in the liver tissues of mice treated with LPS, revealing acute hepatitis. However, PBs treatment markedly decreased the histological alterations in the liver (Additional file 1: Fig. S18). Furthermore, the expression of inflammatory cytokines IL-6 and TNF-α was distinctly reduced after PBs treatment (Fig. 5k, l). The intrinsic ROS-scavenging capability and anti-inflammatory effect of PBs may be responsible for the reduced PTT-induced side effects.

### Self-synergistic effect of PBs in the PTT for cancer

**Fig. 6:**
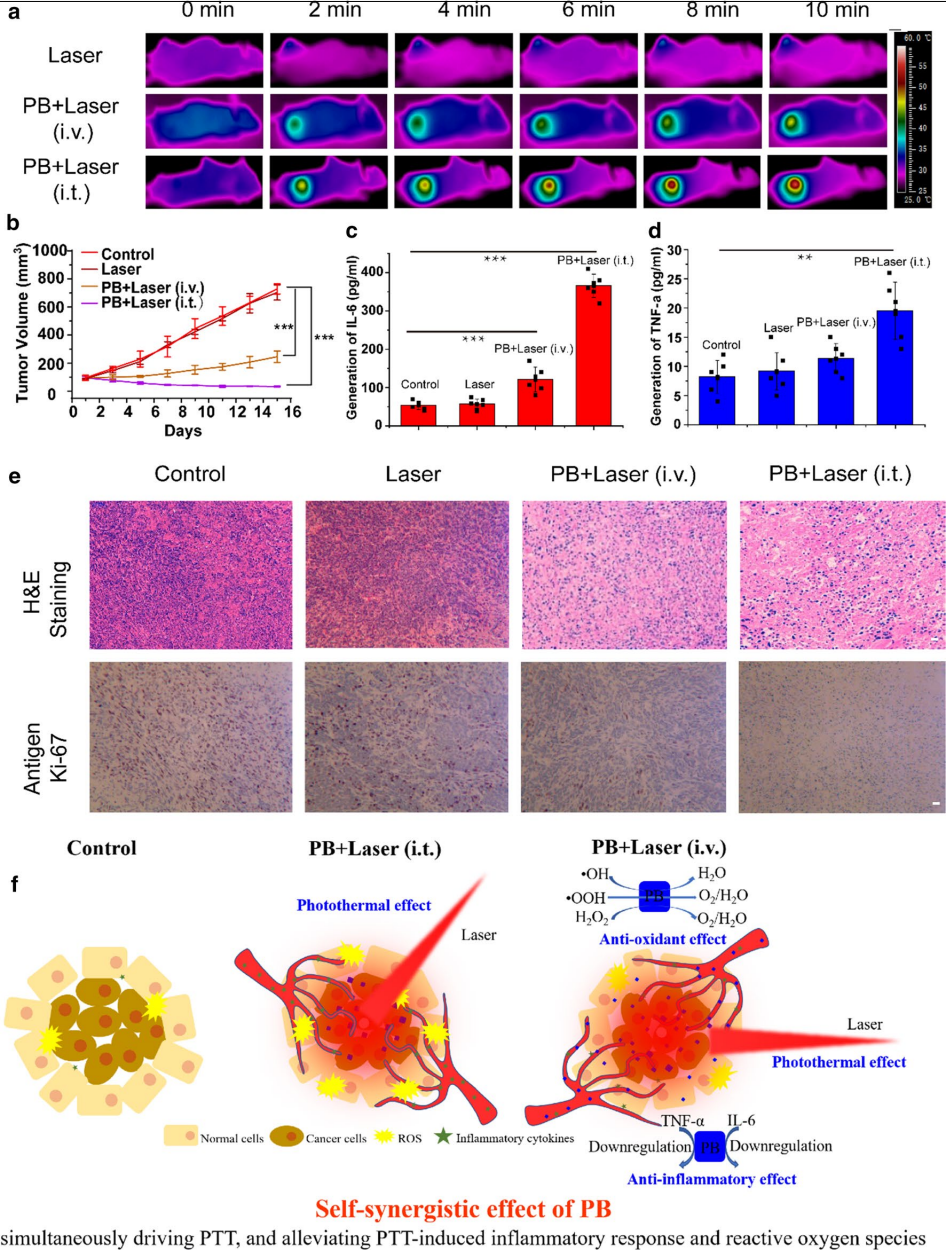
In vivo PTT of PBs in 4T1 tumor-bearing mice. **a** Infrared thermal images of 4T1 tumor-bearing nude mice with different treatments followed by 808 nm laser irradiation (1 W cm^−2^) at different time intervals (0, 2, 4, 6, 8, and 10 min). **b** Time-dependent tumor volume curves of mice in various groups. Levels of **c** IL-6 and **d** TNF-α in the serum after different treatments. **e** H&E staining and Anti-Ki-67 immunofluorescence staining in tumor tissues after different treatments. Scale bar: 50 μm. **f** Intravenous injection of PBs reduced the expression of inflammatory cytokines IL-6 and TNF-α in the serum during PTT. However, intratumor injection of PBs did not stop the PTT-induced increase in the expression of these inflammatory cytokines. Owing to the intrinsic antioxidant and anti-inflammatory properties of PBs, the single-component system could simultaneously drive PTT and alleviate PTT-induced inflammatory response and oxidative stress. (**p < 0.01; ***p < 0.001)

PBs effectively address the problem of inflammatory response and heat stress-induced ROS during PTT owing to their three intrinsic properties of photothermal conversion, ROS scavenging, and anti-inflammation, thereby improving the outcome of PTT and reducing the side effects (inflammatory response and heat stress-induced ROS). As shown in Fig. 6a and Additional file 1: Fig. S19, intravenous administration or intratumor administration of PBs increased the temperature of tumors high enough for ablation. The growth curve of tumor volume with time in each group (Fig. 6b**)** and staining of the tumor sections with H&E and Ki-67 antibody (Fig. 6e**)** demonstrated that the prepared PBs achieved good PTT efficacy under laser irradiation. There were no significant changes in body weight or pathologic changes in major organs (H&E-stained sections), indicating good biosafety (Additional file 1: Figs. S20, S21). The expression of inflammatory cytokines IL-6 and TNF-α in the PBs + Laser (i.t.) group was much higher than those in the control group, Laser group, and PBs + Laser (i.v.) group after 24 h. However, there was no significant difference in their expression between the control group and the PBs + Laser (i.v.) group (Fig. 6c, d). After laser irradiation of the tumor, the PBs induced cancer cell death via apoptosis or necrosis. Intravenous injection of PBs downregulated the expression of inflammatory cytokines IL-6 and TNF-α in the serum. However, the intratumor injection of PBs could not stop the PTT-induced increase in the expression of inflammatory cytokines IL-6 and TNF-α in the serum (Fig. 6c-e). Time-dependent body weight curves of 4T1 tumor-bearing nude mice showed no significant difference in the various groups (Additional file 1: Fig. S20). In addition, H&E staining of tissue sections of major organs from 4T1-bearing nude mice after various treatments displayed no obvious changes, indicating the safety of the prepared PBs (Additional file 1: Fig. S21). Results of the LPS model and xenograft tumor model for PTT define the self-synergistic effect of PBs. This proposed concept of self-synergistic effect provides an efficient strategy for promoting the positive effects of a treatment simultaneously reducing its side effects and encourages the development of single-component treatment systems with intrinsic properties rather than combining multiple complex components into one system.

## Conclusion

In summary, we present a novel concept of ‘self-synergistic effect of nanomaterials’ which means that a single-component nano-system can utilize its intrinsic properties to promote positive effects of a treatment while simultaneously reducing side effects of the treatment. We used PBs as an example, and the designed PBs had good photothermal conversion, ROS-scavenging, and anti-inflammatory properties to achieve PTT while simultaneously alleviating PTT-induced inflammation and ROS generation. This efficient strategy overcomes the problem of inflammatory response and heat stress-induced ROS during PTT. The discovery of self-synergistic effect of PBs may promote their further clinical translation. This concept of using self-synergistic systems may open new avenues in the treatment of cancer or other diseases by encouraging scientists to explore and make use of the intrinsic properties of materials.

## Supplementary Information

**Additional file 1: Fig. S1.**TEM images of PBs with different magnification. **Fig. S2.** FTIR spectra of PBs. **Fig. S3.** Photothermal-heating curves of PBs at elevated concentrations under 808 nm laser irradiation. **Fig. S4.** Photothermal-heating curves of PBs dispersed in aqueous solution irradiated at different power intensity (0.2, 0.4, 0.8 and 1.0 W cm^−2^). **Fig. S5.** In vitro photothermal performance of PBs. **Fig. S6.** Digital photographs and the UV–vis-NIR absorbance of PBs dispersed in pure water and saline. **Fig. S7.** UV–vis-NIR absorbance spectra of PBs dispersions before and after irradiation for 10 min and 30 min by 808 nm Laser, respectively. **Fig. S8.** Viability of 4T1 cells incubated with different concentrations of PBs (0, 25, 50, 100, 200, and 400 µg mL^−1^) for 12, 24, and 48 h. **Fig. S9. a** Viability of 4T1 cells after different treatments. **b** Viability of 4T1 cells after photothermal therapy with different PBs concentrations upon laser irradiation. **c** Viability of 4T1 cells treated with PBs (100 μg mL^−1^) upon laser irradiation at varied power densities for 10 min. **Fig. S10.** CLSM images of 4T1 cells stained by calcein-AM and propidium iodide after different treatments. **Fig. S11. a** UV–vis-NIR and **b** fluorescence spectra of PBs and FITC-labeled PBs. **Fig. S12.** CLSM images of 4T1 cells incubated with FITC-labeled PBs (100 μg mL^−1^) for 0, 1, 2 and 4 h. **Fig. S13.** Viability of RAW 264.7 macrophages incubated with different treatments. **Fig. S14.** Levels of inflammatory cytokines in RAW 264.7 macrophages incubated with different treatments. **Fig. S15.** Flow cytometry analysis of CD3^+^, CD3^+^CD8^+^, and CD3^+^CD4^+^ T cells in the blood. **Fig. S16.** Flow cytometry analysis of CD3^+^, CD3^+^CD8^+^, and CD3^+^CD4^+^ T cells in the liver. **Fig. S17.** Flow cytometry analysis of CD3^+^, CD3^+^CD8^+^, and CD3^+^CD4^+^ T cells in the spleen. **Fig. S18.** The hematoxylin and eosin staining of liver in various groups. **Fig. S19.** Temperature curves at the tumor region of 4T1-tumor-bearing nude mice in different groups under 808 nm laser irradiation for 10 min. **Fig. S20.** Time-dependent body-weight curves of 4T1 tumor-bearing nude mice. **Fig. S21.** H&E-staining tissue of major organs.

## Data Availability

The datasets and materials used in the study are available from the corresponding author.
