# Supplementary material for: Self-synergistic effect of Prussian blue nanoparticles for cancer therapy: driving photothermal therapy and reducing hyperthermia-induced side effects
Source: J Nanobiotechnology. 2021 May 4;19:126. doi: 10.1186/s12951-021-00819-2 (PMC8098002; doi:10.1186/s12951-021-00819-2)
Supplement: Supplementary file 1 — Additional file 1: Fig. S1.TEM images of PBs with different magnification. Fig. S2. FTIR spectra of PBs. Fig. S3. Photothermal-heating curves of PBs at elevated concentrations under 808 nm laser irradiation. Fig. S4. Photothermal-heating curves of PBs dispersed in aqueous solution irradiated at different power intensity (0.2, 0.4, 0.8 and 1.0 W cm−2). Fig. S5. In vitro photothermal performance of PBs. Fig. S6. Digital photographs and the UV–vis-NIR absorbance of PBs dispersed in pure water and saline. Fig. S7. UV–vis-NIR absorbance spectra of PBs dispersions before and after irradiation for 10 min and 30 min by 808 nm Laser, respectively. Fig. S8. Viability of 4T1 cells incubated with different concentrations of PBs (0, 25, 50, 100, 200, and 400 µg mL−1) for 12, 24, and 48 h. Fig. S9. a Viability of 4T1 cells after different treatments. b Viability of 4T1 cells after photothermal therapy with different PBs concentrations upon laser irradiation. c Viability of 4T1 cells treated with PBs (100 μg mL−1) upon laser irradiation at varied power densities for 10 min. Fig. S10. CLSM images of 4T1 cells stained by calcein-AM and propidium iodide after different treatments. Fig. S11. a UV–vis-NIR and b fluorescence spectra of PBs and FITC-labeled PBs. Fig. S12. CLSM images of 4T1 cells incubated with FITC-labeled PBs (100 μg mL−1) for 0, 1, 2 and 4 h. Fig. S13. Viability of RAW 264.7 macrophages incubated with different treatments. Fig. S14. Levels of inflammatory cytokines in RAW 264.7 macrophages incubated with different treatments. Fig. S15. Flow cytometry analysis of CD3+, CD3+CD8+, and CD3+CD4+ T cells in the blood. Fig. S16. Flow cytometry analysis of CD3+, CD3+CD8+, and CD3+CD4+ T cells in the liver. Fig. S17. Flow cytometry analysis of CD3+, CD3+CD8+, and CD3+CD4+ T cells in the spleen. Fig. S18. The hematoxylin and eosin staining of liver in various groups. Fig. S19. Temperature curves at the tumor region of 4T1-tumor-bearing nude mice in different groups under 808 nm [file 12951_2021_819_MOESM1_ESM.docx]

Supporting Information

Self-synergistic effect of Prussian blue nanoparticles for cancer therapy: driving photothermal therapy and reducing hyperthermia-induced side effects

Xue Xie ^1,2†^, Wei Gao^1†^, Junnian Hao^1^, Jianrong Wu^1*^, Xiaojun Cai^1*^, Yuanyi Zheng^1*^

*Correspondence: wujr_028@126.com; c1x2j34@163.com; zhengyuanyi@sjtu.edu.cn

^†^ Xue Xie and Wei Gao contributed equally to this work

1. Shanghai Institute of Ultrasound in Medicine, Shanghai Jiao Tong University Affiliated Sixth People's Hospital, Shanghai 200233, P.R. China.

2. Chongqing Key Laboratory of Ultrasound Molecular Imaging, Ultrasound Department of the Second Affiliated Hospital of Chongqing Medical University. Chongqing 400010, People’s Republic of China


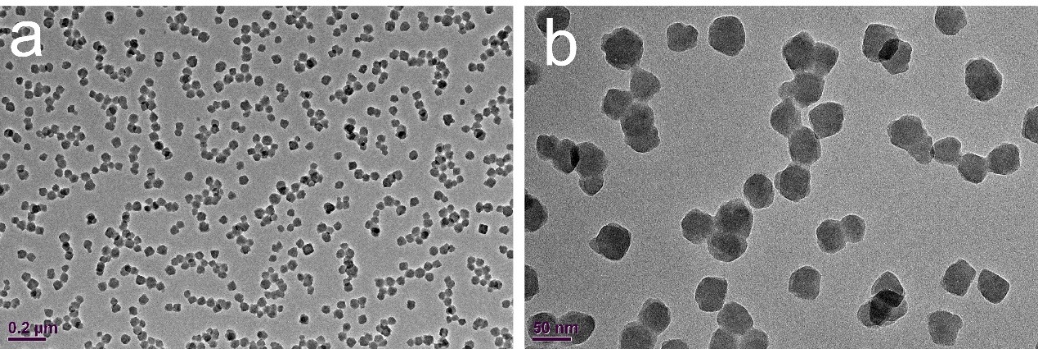


**Figure S1.**TEM images of PBs with different magnification.


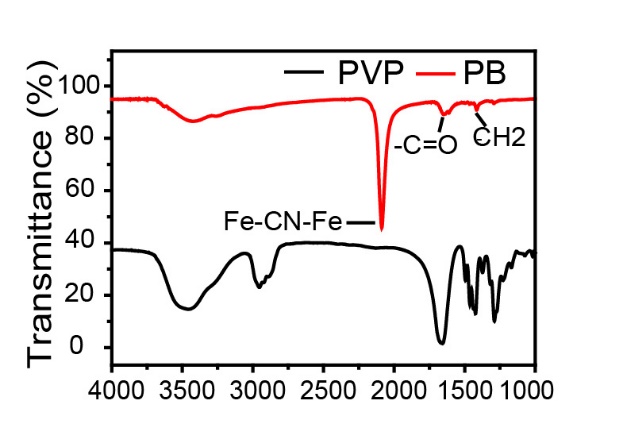


**Figure S2.** FTIR spectra of PBs. FTIR displays a characteristic peak around 2085 cm^-1^ of Fe^2+^-CN-Fe^3+^, the absorption peak at 1670 cm^-1^ is characteristic of the C=O group of PVP.


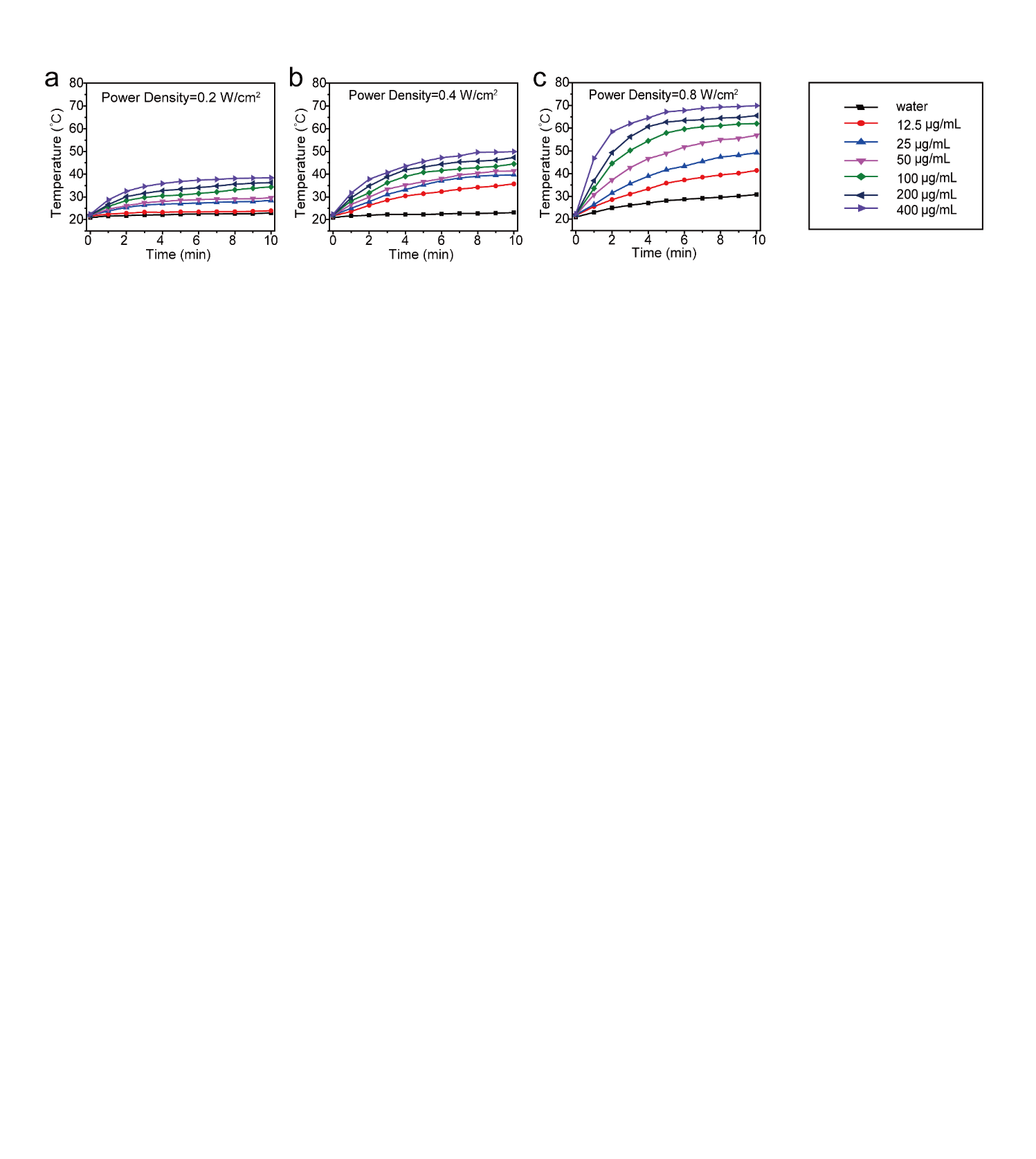


**Figure S3.** Photothermal-heating curves of PBs at elevated concentrations under 808 nm laser irradiation. (a) 0.2 W cm^-2^. (b) 0.4 W cm^-2^ and (c) 0.8 W cm^-2^.


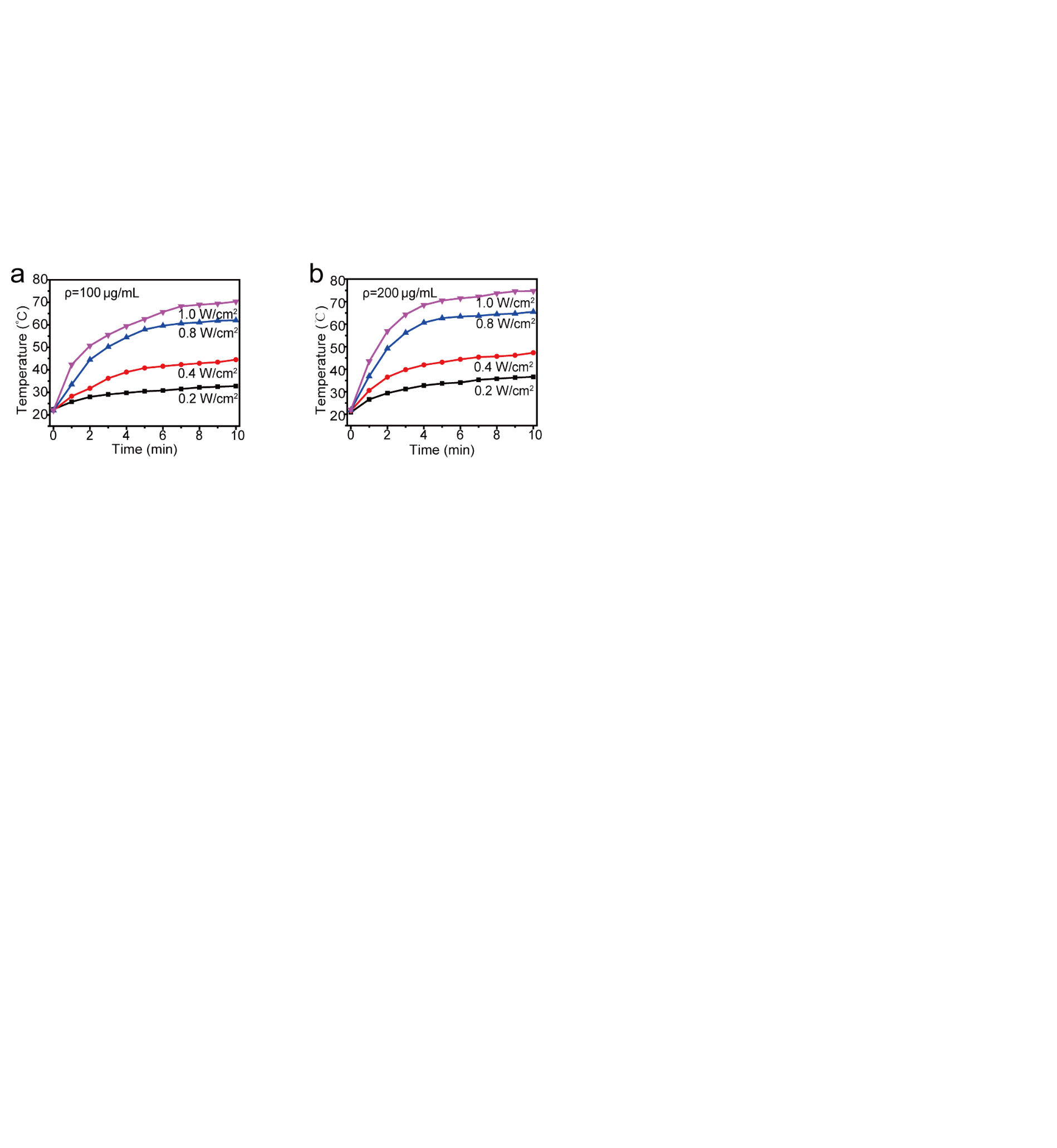


**Figure S4.** Photothermal-heating curves of PBs dispersed in aqueous solution irradiated at different power intensity (0.2, 0.4, 0.8 and 1.0 W cm^-2^). (a) PB concentration (100 μg mL^-1^). (b) PB concentration (200 μg mL^-1^).


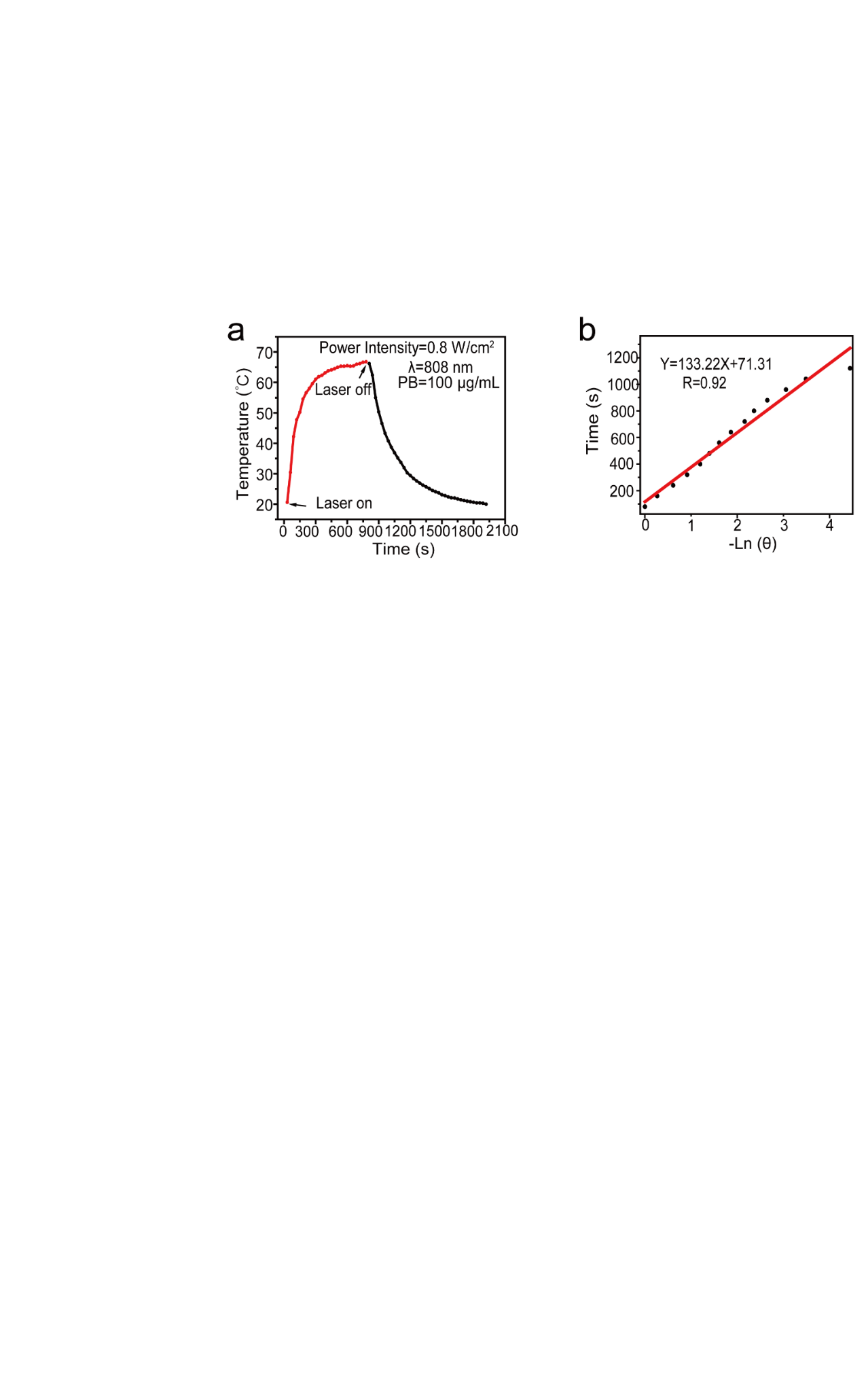


**Figure S5.** In vitro photothermal performance of PBs. (a) Photothermal performance of the PBs under irradiation by a NIR laser( 808 nm, 0.8 W cm^-2^) .(b) Time constant for heat transfer from the system was determined to be 133.22 s by applying the linear time data from the cooling period versus negative natural logarithm of driving force temperature, which was originated from the cooling stage.


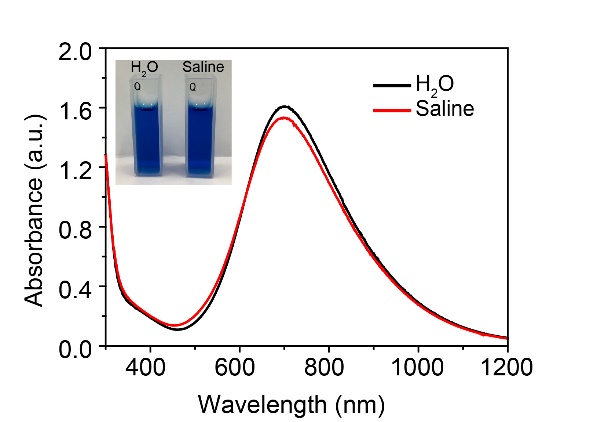


**Figure S6.** Digital photographs and the UV-vis-NIR absorbance of PBs dispersed in pure water and saline.


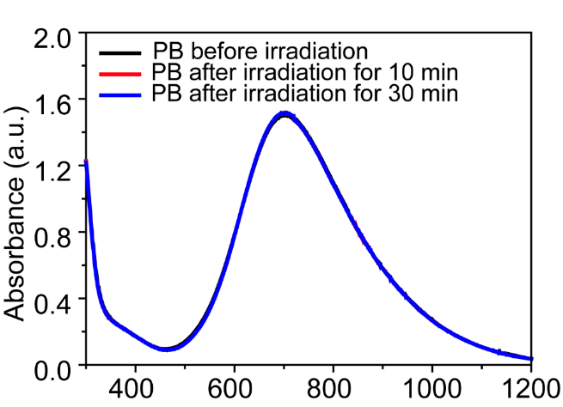


**Figure S7.** UV-vis-NIR absorbance spectra of PB dispersions before and after irradiation for 10 min and 30 min by 808 nm laser, respectively.


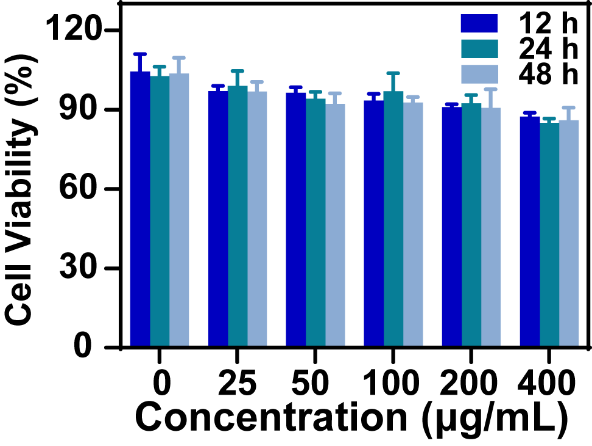


**Figure S8.**Viability of 4T1 cells incubated with different concentrations of PBs (0, 25, 50, 100, 200, and 400 µg mL^-1^) for 12, 24, and 48 h.


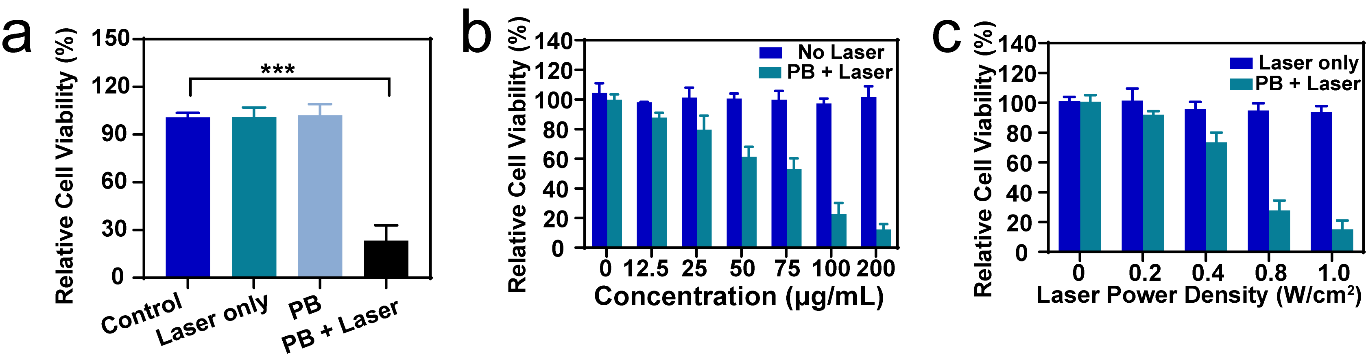
 **Figure S9. a** Viability of 4T1 cells after different treatments. Error bars represent the standard deviation of five parallel samples (*** p < 0.001). **b** Viability of 4T1 cells after photothermal therapy with different PBs concentrations (0, 12.5, 25, 50, 75, 100, 200 μg mL^-1^) upon laser irradiation (1.0 W cm^-2^, 10 min). **c** Viability of 4T1 cells treated with PB (100 μg mL^-1^) upon laser irradiation at varied power densities (0, 0.2, 0.4, 0.8, 1.0 W cm^-2^) for 10 min.


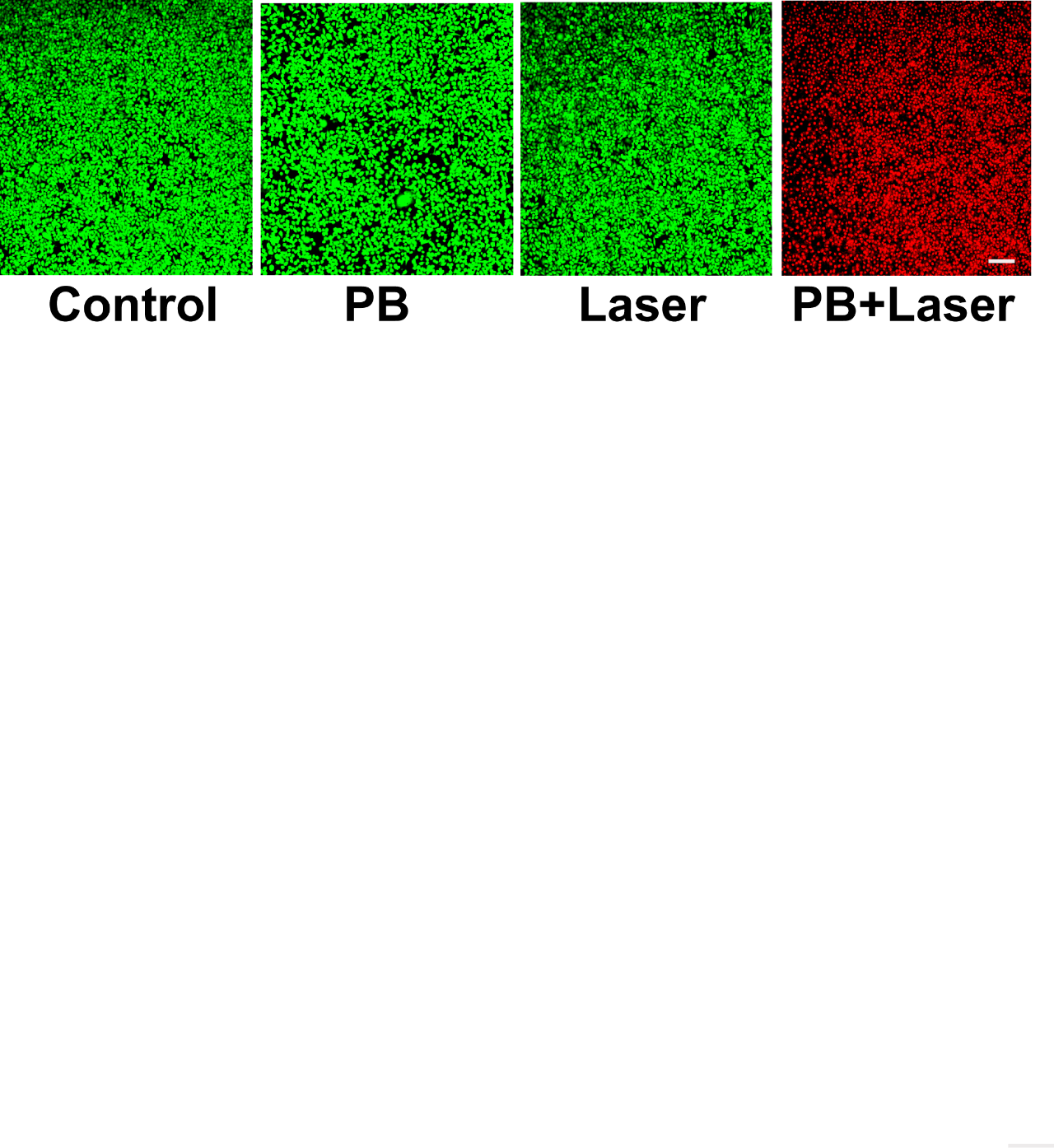


**Figure S10.** CLSM images of 4T1 cells stained by calcein-AM (green; living cells) and propidium iodide (PI; red; dead cells) after different treatments. (Scale bars: 50 µm).


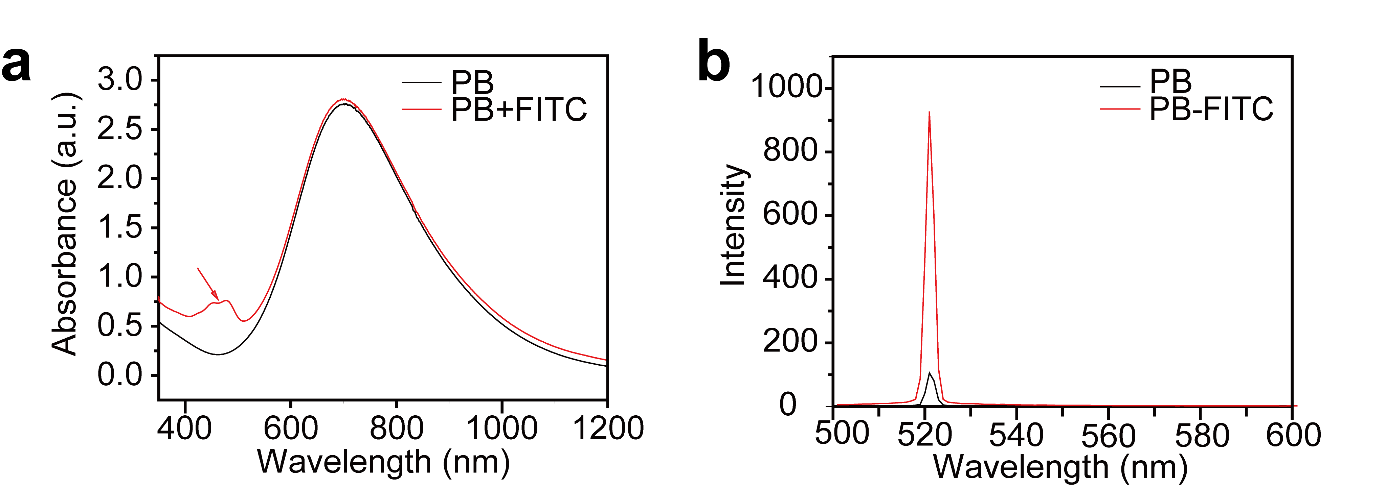


**Figure S11. a** UV-vis-NIR and **b** fluorescence spectra of PB and FITC-labeled PB.


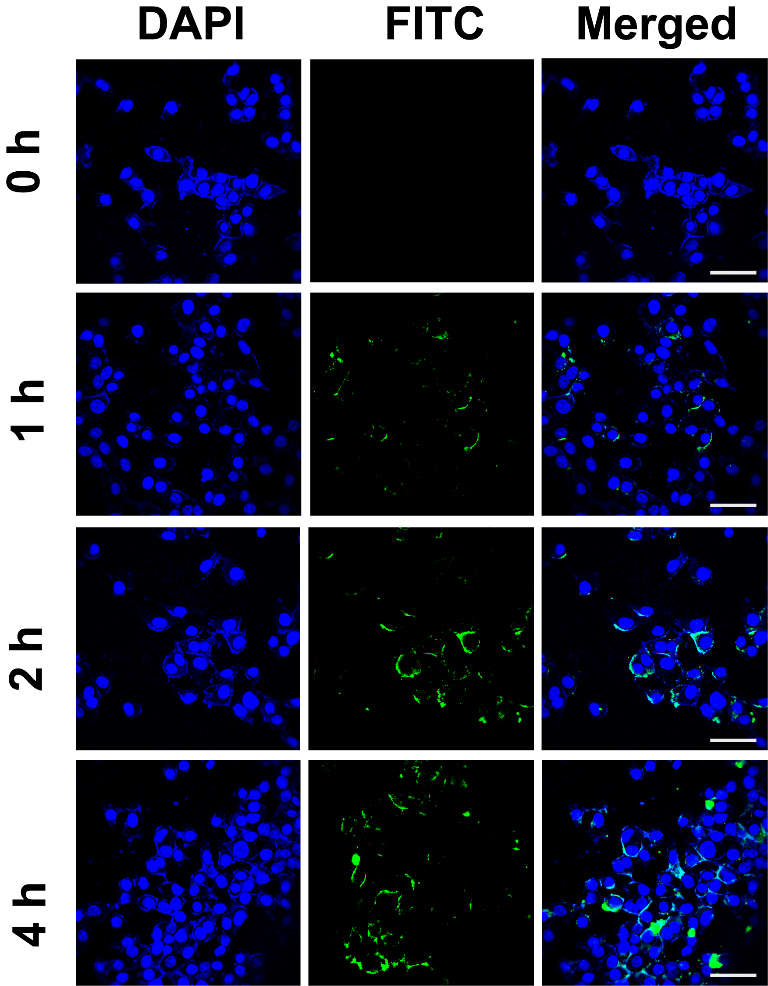


**Figure S12.** CLSM images of 4T1 cells incubated with FITC-labeled PB (100 μg mL^-1^) for 0, 1, 2 and 4 h. The nuclei stained blue with DAPI, and the PBs stained green with FITC under CLSM. Scale bar: 50 µm.


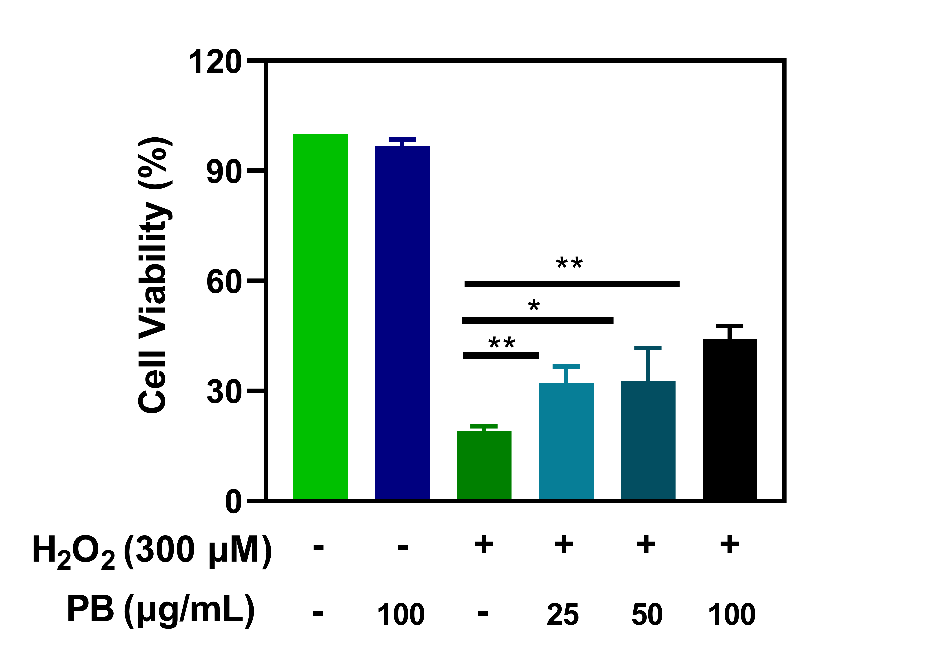


**Figure S13.** Viability of RAW 264.7 macrophages incubated with different treatments. (*p < 0.05; **p < 0.01).


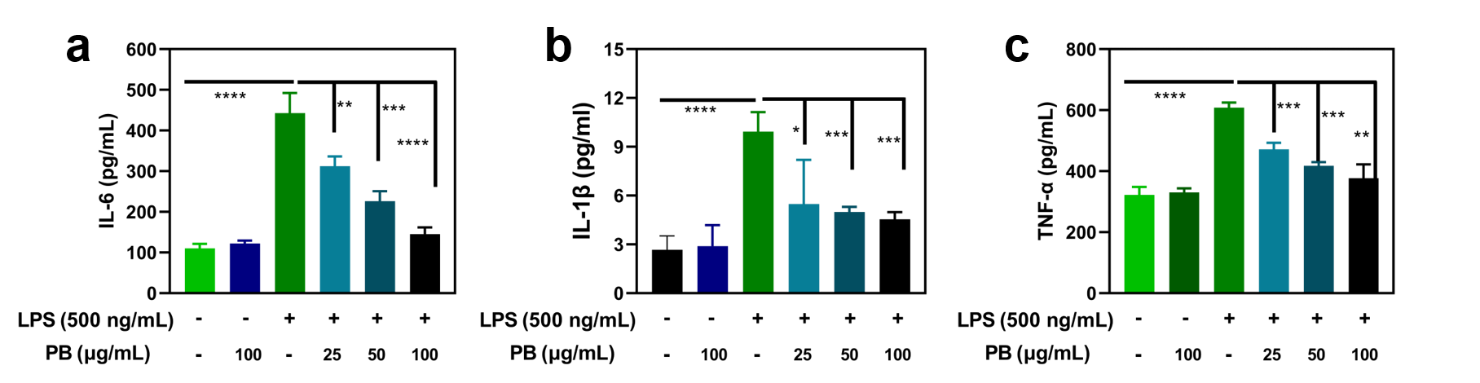


**Figure S14.** Levels of inflammatory cytokines in RAW 264.7 macrophages incubated with different treatments. **a** IL-6, **b** IL-1β and **c** TNF-α. (*, p < 0.05; **, p < 0.01; ***, p < 0.001; ****; p < 0.0001).


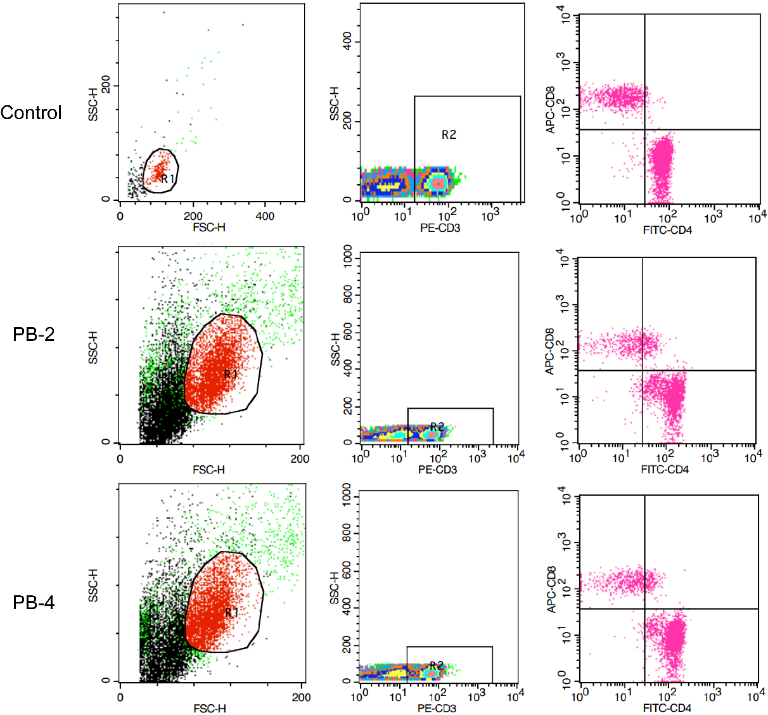


**Figure S15.** Flow cytometry analysis of CD3^+^, CD3^+^CD8^+^, and CD3^+^CD4^+^ T cells in the blood.


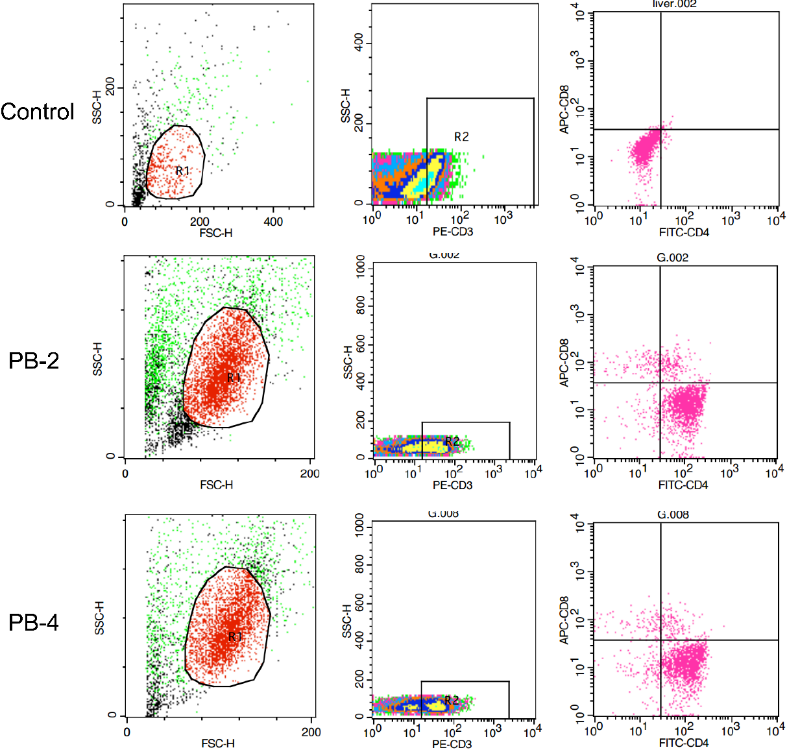


**Figure S16.** Flow cytometry analysis of CD3^+^, CD3^+^CD8^+^, and CD3^+^CD4^+^ T cells in the liver.


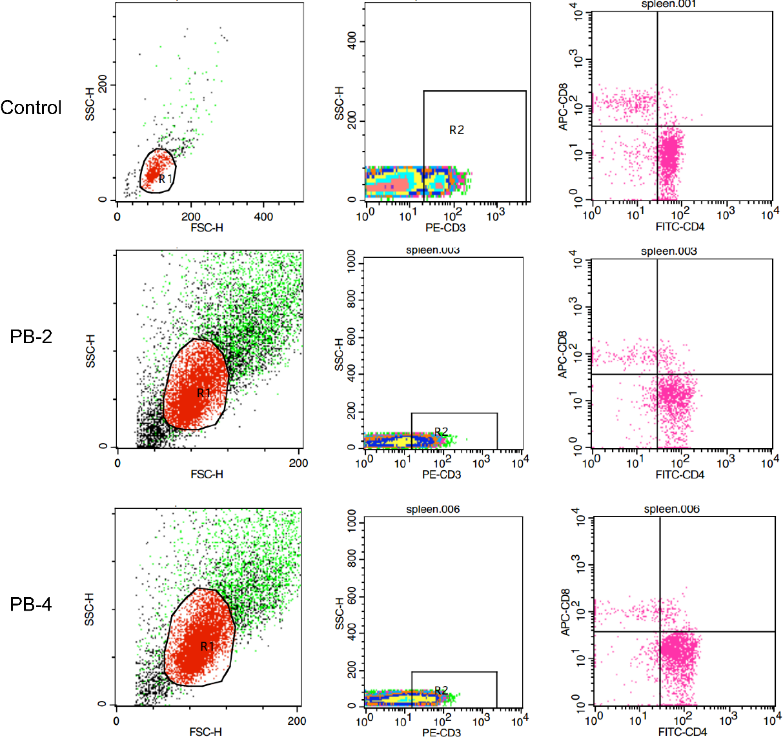


**Figure S17.** Flow cytometry analysis of CD3^+^, CD3^+^CD8^+^, and CD3^+^CD4^+^ T cells in the spleen.


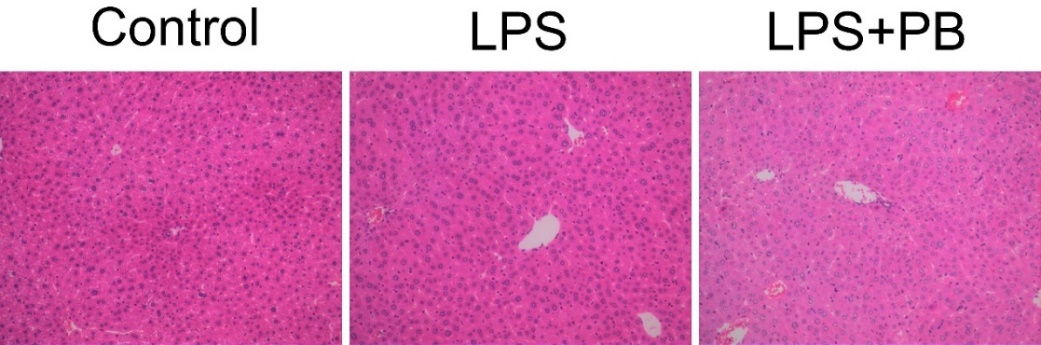


**Figure S18.** The hematoxylin and eosin staining of liver in various groups. The focal nuclear pyknosis, inflammatory cell infiltration, and even bile stasis could be observed in the livers of mice treated with LPS. While with PBs treatment, the livers display markedly decreased histological alterations.


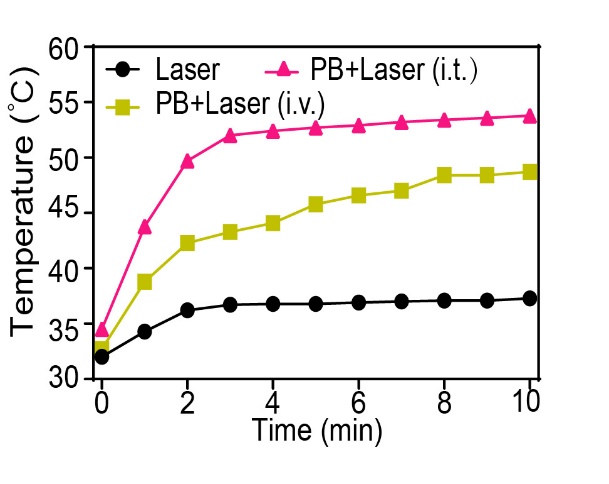


**Figure S19.** Temperature curves at the tumor region of 4T1-tumor-bearing nude mice in different groups under 808 nm laser irradiation (The laser density=1.0 W cm^-2^) for 10 min.

**
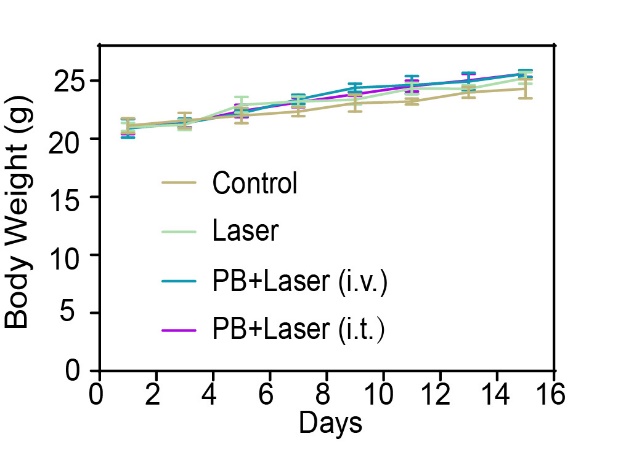
**

**Figure S20.** Time-dependent body-weight curves of 4T1 tumor-bearing nude mice.


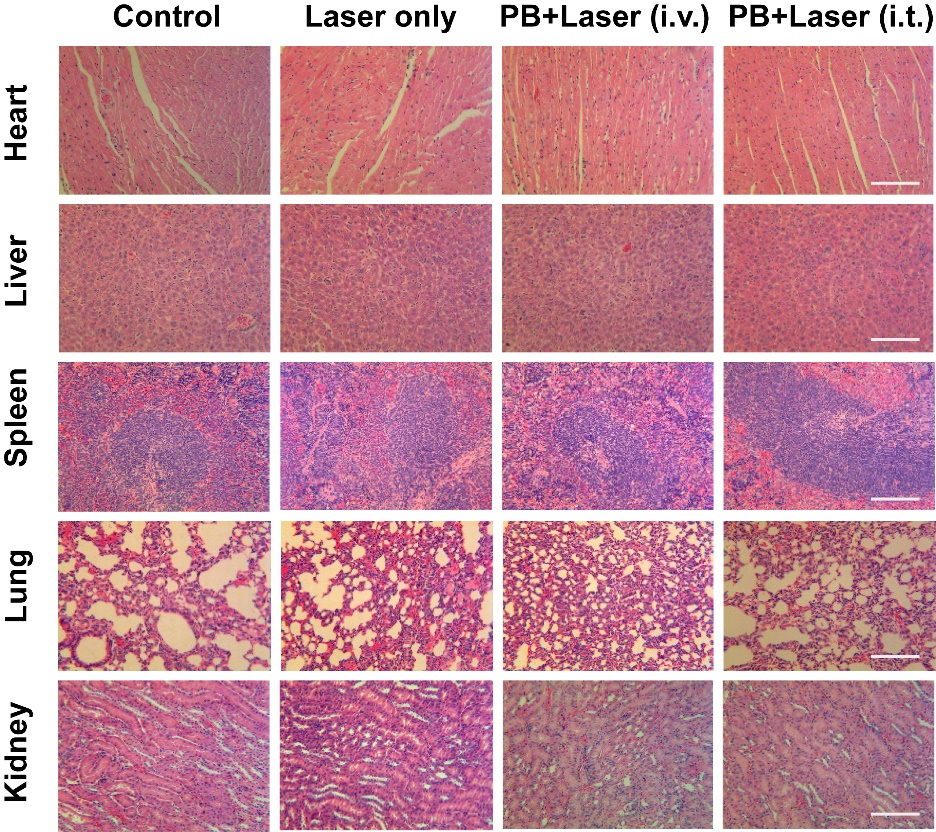


**Figure S21.** H&E-staining tissue of major organs from 4T1-bearing nude mice after various treatments. All the scale bars are 50 μm.
